# A tool for nuclear imaging of the SARS-CoV-2 entry receptor: molecular model and preclinical development of ACE2-selective radiopeptides

**DOI:** 10.1186/s13550-023-00979-2

**Published:** 2023-04-19

**Authors:** Darja Beyer, Christian Vaccarin, Xavier Deupi, Ana Katrina Mapanao, Susan Cohrs, Fan Sozzi-Guo, Pascal V. Grundler, Nicholas P. van der Meulen, Jinling Wang, Matthias Tanriver, Jeffrey W. Bode, Roger Schibli, Cristina Müller

**Affiliations:** 1 Center for Radiopharmaceutical Sciences, ETH-PSI, Paul Scherrer Institute, 5232 Villigen-PSI, Switzerland; 2 Condensed Matter Theory Group, Division of Scientific Computing, Theory, and Data, Paul Scherrer Institute, 5232 Villigen-PSI, Switzerland; 3 Laboratory of Biomolecular Research, Paul Scherrer Institute, 5232 Villigen-PSI, Switzerland; 4 Swiss Institute of Bioinformatics (SIB), Lausanne, Switzerland; 5 Laboratory of Radiochemistry, Paul Scherrer Institute, 5232 Villigen-PSI, Switzerland; 6 Institute of Organic Chemistry, Department of Chemistry and Applied Biosciences, ETH Zurich, 8093 Zurich, Switzerland; 7 Institute of Pharmaceutical Sciences, Department of Chemistry and Applied Biosciences, ETH Zurich, 8093 Zurich, Switzerland

**Keywords:** DX600, Radiopeptide, Gallium, PET, ACE2, ACE, SARS-CoV-2

## Abstract

**Purpose:**

The angiotensin converting enzyme-2 (ACE2)—entry receptor of SARS-CoV-2—and its homologue, the angiotensin-converting enzyme (ACE), play a pivotal role in maintaining cardiovascular homeostasis. Potential changes in ACE2 expression levels and dynamics after SARS-CoV-2 infection have been barely investigated. The aim of this study was to develop an ACE2-targeting imaging agent as a noninvasive imaging tool to determine ACE2 regulation.

**Methods:**

DOTA-DX600, NODAGA-DX600 and HBED-CC-DX600 were obtained through custom synthesis and labeled with gallium-67 (*T*_1/2_ = 3.26 d) as a surrogate radioisotope for gallium-68 (*T*_1/2_ = 68 min). ACE2- and ACE-transfected HEK cells were used for the in vitro evaluation of these radiopeptides. The in vivo tissue distribution profiles of the radiopeptides were assessed in HEK-ACE2 and HEK-ACE xenografted mice and imaging studies were performed using SPECT/CT.

**Results:**

The highest molar activity was obtained for [^67^Ga]Ga-HBED-CC-DX600 (60 MBq/nmol), whereas the labeling efficiency of the other peptides was considerably lower (20 MBq/nmol). The radiopeptides were stable over 24 h in saline (> 99% intact peptide). All radiopeptides showed uptake in HEK-ACE2 cells (36–43%) with moderate ACE2-binding affinity (*K*_D_ value: 83–113 nM), but no uptake in HEK-ACE cells (< 0.1%) was observed. Accumulation of the radiopeptides was observed in HEK-ACE2 xenografts (11–16% IA/g) at 3 h after injection, but only background signals were seen in HEK-ACE xenografts (< 0.5% IA/g). Renal retention was still high 3 h after injection of [^67^Ga]Ga-DOTA-DX600 and [^67^Ga]Ga-NODAGA-DX600 (~ 24% IA/g), but much lower for [^67^Ga]Ga-HBED-CC-DX600 (7.2 ± 2.2% IA/g). SPECT/CT imaging studies confirmed the most favorable target-to-nontarget ratio for [^67^Ga]Ga-HBED-CC-DX600.

**Conclusions:**

This study demonstrated ACE2 selectivity for all radiopeptides. [^67^Ga]Ga-HBED-CC-DX600 was revealed as the most promising candidate due to its favorable tissue distribution profile. Importantly, the HBED-CC chelator enabled ^67^Ga-labeling at high molar activity, which would be essential to obtain images with high signal-to-background contrast to detect (patho)physiological ACE2 expression levels in patients.

**Supplementary Information:**

The online version contains supplementary material available at 10.1186/s13550-023-00979-2.

## Introduction

Covid-19, an infectious disease caused by the SARS-CoV-2 virus, was declared a pandemic by the World Health Organization in March 2020 and the disease has become a significant global health threat to society [1]. The numerous virus variants have contributed to variable pathological manifestations, symptom severity and long-term effects of Covid-19 [2]. SARS-CoV-2 uses the angiotensin converting enzyme-2 (ACE2) as an entry receptor for host cell infection [3]. ACE2 was discovered in the year 2000 and identified as an essential enzyme for cardiovascular regulation processes [4, 5]. The catalytic domain of ACE2 shares 42% sequence identity with its homologue, the angiotensin-converting enzyme (ACE) [4]; however, these enzymes differ in terms of substrate selectivity and physiological functions [6, 7]. Importantly, ACE2 does not bind ACE inhibitors such as captopril or lisinopril, which are part of a frequently used class of drugs to treat hypertension [8]. ACE2 has an antihypertensive and cardioprotective role and acts as counter regulator of ACE in the renin–angiotensin–aldosterone system (RAAS). ACE2 was found to be highly expressed in the cardiovascular system, lungs, kidneys, intestines and testis. Not surprisingly, several of these tissues were identified as critical sites in Covid-19 pathological manifestations [9, 10].

The complex regulation mechanisms of ACE2 expression, including dynamic changes after SARS-CoV-2 infection, may be one of several reasons why Covid-19 displays such large variability of manifestations and progression ranging from asymptomatic to severe cases with fatal outcomes [11, 12]. Some studies propose a positive correlation between ACE2 expression and disease severity as a result of enhanced viral propagation [13], and argue that lower ACE2 levels in children protects them from severe SARS-CoV-2 infections [14]. Other studies suggest a poor prognosis of elderly as a result of low ACE2 expression. The contradictory statements with regard to age-related ACE2 expression [15–17] may originate from strong inter-individual variation, cell-type-dependent maximum age of ACE2 degradation and the fact that mRNA and protein levels do not exactly correspond [14]. There is also the theory that the degradation of ACE2 through infection with SARS-CoV-2 and, thus, the lack in ability to counterbalance the action of ACE in the RAAS may lead to a pro-inflammatory state and, therefore, an increased risk of a severe disease progression [14].

The hypothesis that ACE2 expression levels and dynamics affects Covid-19 severity is further supported by the observation that medical conditions that can affect the ACE2 expression levels such as hypertension, diabetes and obesity, but also genetic predisposition and certain medications, are known risk factors for severe disease phenotypes of Covid-19 [18].

A better understanding of the dynamics of ACE2 expression as a means to predict the susceptibility to infection and disease progression would possibly enable identifying patients at risk and allow the development of more individualized treatment strategies. While attempts have been made to determine ACE2 in various tissues at the mRNA and protein levels [19–21], noninvasive imaging would also enable to determine the expression dynamics directly in patients.

Among the imaging techniques used in the clinic, single photon emission computed tomography (SPECT) is still more often employed than positron emission tomography (PET) [22]. The enhanced image resolution, the increased sensitivity and the option for accurate quantification present, however, relevant advantages of PET, which would, therefore, be preferred for molecular imaging of (patho)physiological processes of many medical situations, including cardiovascular diseases [23]. Preclinical attempts to develop a PET imaging agent to visualize ACE2 were initially made by labeling the receptor-binding domain of the viral spike protein with iodine-124 [24]. Other research groups used the disulfide-bridged DX600, initially developed as an ACE2-specific inhibitor [25], for derivatization with a DOTA or NODAGA chelator to enable coordination of gallium-68 as a PET radionuclide [26, 27]. A NOTA-derivatized DX600 peptide, referred to as DX600-BCH, was developed for coordination of aluminium-fluoride-18 and is currently being used in a clinical trial to test the possibility of noninvasive mapping of ACE2 (NCT04542863) [28].

**Fig. 1:**
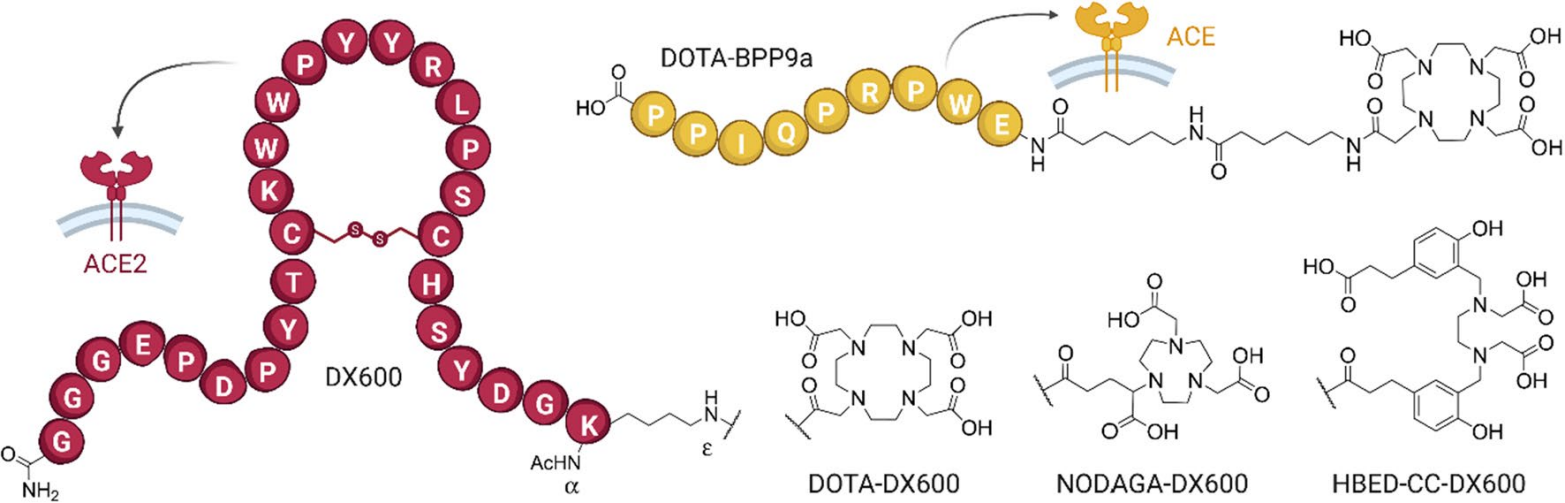
Schematic structures of DOTA-DX600, NODAGA-DX600, HBED-CC-DX600 (red) for targeting ACE2 and DOTA-BPP9a (yellow) for targeting ACE

Our aim was to perform a more in-depth investigation of DX600-based radiopeptides for noninvasive PET imaging of ACE2. With the aid of molecular modeling, three peptides with a DOTA, a NODAGA or a HBED-CC chelator, respectively, were designed (Fig. 1) [29]. The three DX600 peptides were evaluated after labeling with gallium-67 (*T*_1/2_ = 3.26 d), as a surrogate radioisotope for gallium-68 (*T*_1/2_ = 68 min), due to its more convenient half-life and because it can be used for SPECT. The ACE2 selectivity of the DX600-based radiopeptides was assessed by the use of ACE2- and ACE-transfected human embryonic kidney (HEK) cells and respective xenograft mouse models. An ACE-specific radiopeptide based on the structure of the bradykinin-potentiating peptide-9a (BPP9a [30]) was synthesized and derivatized for metal chelation for the purpose of validating the in vitro and in vivo models (Fig. 1).

## Methods

### Three‐dimensional molecular model of the ACE2/DX600 complex

The 3D-structure of the DX600 peptide in water has been solved by NMR [31]. The resulting conformational ensemble showed that the constraints imposed by the intramolecular disulfide bridge leads to the formation of a central closed structured loop, while the C and N termini have a predominantly disordered structure. The structured loop contains a consensus inhibitory motif (CxPxRxxPWxxC) [25] that is likely to interact with the buried catalytic residues at the core of the catalytic domain of ACE2. The structure of human ACE2 bound to the small-molecular-weight inhibitor MLN-4760 [32] was used as a template to model the complex with the DX600 peptide using PyMOL (The PyMOL Molecular Graphics System, Version 2.5.2, Schrödinger, LLC). This model was subsequently refined using the Haddock v2.4 software [33]. Finally, an N-α-acetyl-lysine residue coupled to a gallium-coordinating HBED-CC chelator on the ε-amino group was fused to Gly1 in DX600 using the crystal structure of HBED-CC bound to the nickel-binding periplasmic protein as a template.

### ACE2- and ACE-targeting peptides

The ACE2-targeting peptides were obtained as custom synthesis from piCHEM (Research & Development GmbH, Raaba-Grambach, Austria). The cyclic DX600 peptides were functionalized with an *N*-*α*-acetyl-lysine residue at the N terminus. Subsequently, a DOTA, NODAGA or HBED-CC chelator, respectively, was coupled to the *N* terminus at the *ε*-amino group present on the lysine sidechain to obtain DOTA-DX600, NODAGA-DX600 and HBED-CC-DX600 (Fig. 1; Additional file 1: Table S1). The chemical identity and purity (> 95%) of the purchased peptides were confirmed by HR-MS (MALDI-TOF) and analytical HPLC, respectively (Additional file 1: Fig. S1, Table S2). The unmodified DX600 peptide, herein referred to as cyclo-DX600 (piCHEM, Research & Development GmbH, Raaba-Grambach, Austria), was used for in vitro experiments to block ACE2.

An ACE-targeting agent was designed based on the BPP9a peptide as a lead structure [34]. In brief, the immobilized and side chain-protected BPP9a peptide was produced by standard Fmoc solid-phase peptide chemistry. Afterward, the peptide was functionalized with a spacer constituted of two consecutive 6-aminohexanoic residues at the N terminus. The resulting N-terminal amino group was coupled with a DOTA-tris(^*t*^Bu) ester. Acid-catalyzed cleavage from the resin led to complete removal of the protecting groups (Additional file 1: Scheme S1). The crude peptide was purified using semipreparative HPLC, resulting in moderate yields (19%) of the desired DOTA-BPP9a (Fig. 1). The chemical identity and purity of the peptide were confirmed by HRMS and HPLC analyses, respectively (Additional file 1: Fig. S2).

### Post-purification of gallium-67

Gallium-67 (no-carrier-added [^67^Ga]GaCl_3_ in ~ 0.1 M HCl) was purchased from Curium Netherlands B.V., the Netherlands, via b.e.imaging GmbH (Switzerland). A post-delivery process of the purchased gallium-67 was necessary to enable radiolabeling at the indicated molar activities. This was carried out according to a previously published method [35]. In brief, other trivalent transition metals were selectively reduced by addition of TiCl_3_ and, subsequently, the desired [^67^Ga]GaCl_3_ was isolated by absorption chromatography performed on Amberchrom CG-161M resin. An extraction resin was used for concentration of the [^67^Ga]GaCl_3_ in a small volume of dilute HCl.

### Preparation of the radiopeptides and stability of the radiopeptide formulation

The purified [^67^Ga]GaCl_3_ in HCl (~ 0.1 M) was added to a sodium acetate solution (0.5 M, pH 8) to obtain a buffered system of pH ~ 4. After adding the respective peptide (DOTA-DX600, NODAGA-DX600, HBED-CC-DX600 or DOTA-BPP9a) in a quantity to obtain the desired molar activity (5–20 MBq/nmol), the reaction mixture was incubated at 95 °C for 15 min. Quality control was performed using a Merck Hitachi LaChrom HPLC system (Additional file 1). The radiopeptides were used directly for in vitro and in vivo studies without further purification steps. The stability of the radiopeptides (20 MBq/nmol) formulated in 0.9% NaCl (10 MBq/100 µL) was assessed at room temperature (Additional file 1). The highest molar activity used in these studies was 20 MBq/nmol which means that one of 73–74 molecules was labeled with gallium-67. Nevertheless, the labeling efficiency of the DX600-based peptides was assessed using the highest possible molar activity that would still allow for the preparation of the DX600-based radiopeptide at a radiochemical purity of > 95% (Additional file 1).

### Distribution coefficients of the radiopeptides

The distribution coefficients (logD values) of [^67^Ga]Ga-DOTA-DX600, [^67^Ga]Ga-NODAGA-DX600, [^67^Ga]Ga-HBED-CC-DX600 and [^67^Ga]Ga-DOTA-BPP9a were determined by the shake flask method. An aliquot of the respective radiopeptides (0.5 MBq; 25 pmol, 25 µL) was added to a vial containing a mixture of phosphate buffered saline (PBS) pH 7.4 (1475 µL) and n-octanol (1500 µL). After vortexing vigorously for 1 min, the vials were centrifuged (560 rcf; 6 min) to obtain phase separation. The quantity of activity in the organic and aqueous phases was determined in a *γ*-counter (PerkinElmer, Wallac Wizard 1480). The distribution coefficients were expressed as the logarithm of the ratio of counts per minute (cpm) measured in the n-octanol phase to the cpm measured in the PBS phase and indicated as the average of three independent measurements (± standard deviation, SD), each performed with five replicates.

### Blood plasma protein-binding properties

The radiopeptides (0.3 MBq; 15 pmol) were incubated in human or murine blood plasma (150 μL) for 30 min at 37 °C followed by cooling on ice, addition of ice-cold PBS (150 μL) and transfer of the samples to amicon filter devices (Amicon Ultra, 0.5 mL, Merck Millipore; cutoff 10 kDa). The filter ensured the retention of the radiopeptides bound to mouse plasma proteins or human plasma proteins during filtration (14,000 rcf, 30 min at 4 °C). Afterward, the filter inserts were inverted and centrifuged again (200 rcf, 3 min) to recover the protein-bound fraction of the radiopeptides. The activities of the free radiopeptides in the filtrate, as well as in the filter insert and the plasma protein-bound radiopeptides (*A*_bound_), were counted for activity separately in a *γ*-counter. The total activity (*A*_total_) was determined as the sum of the activities in the filtrate, filter insert, and protein-bound fractions. The percentage of radiopeptide bound to mouse serum albumin (MSA) and human serum albumin (HSA) was calculated as *A*_bound_/*A*_total_*100. The results were presented as average ± SD of three independent experiments (Additional file 1).

### Cell culture

HEK-293 cells transfected with ACE2 or ACE, referred to as HEK-ACE2 and HEK-ACE, respectively, were obtained from Innoprot (Innovative Technologies in Biological Systems S.L. Bizkaia, Spain). The cells were cultured in Dulbecco’s Modified Eagle Medium (DMEM) supplemented with nonessential amino acids, fetal calf serum and antibiotics. Hygromycin B was used to maintain the expression of ACE2 and ACE, respectively. The HEK cells were cultured under standard conditions at 37 °C and 5% CO_2_ and subcultured using PBS/EDTA and trypsin.

### Validation of ACE2 and ACE expression in HEK cells

**Fig. 2:**
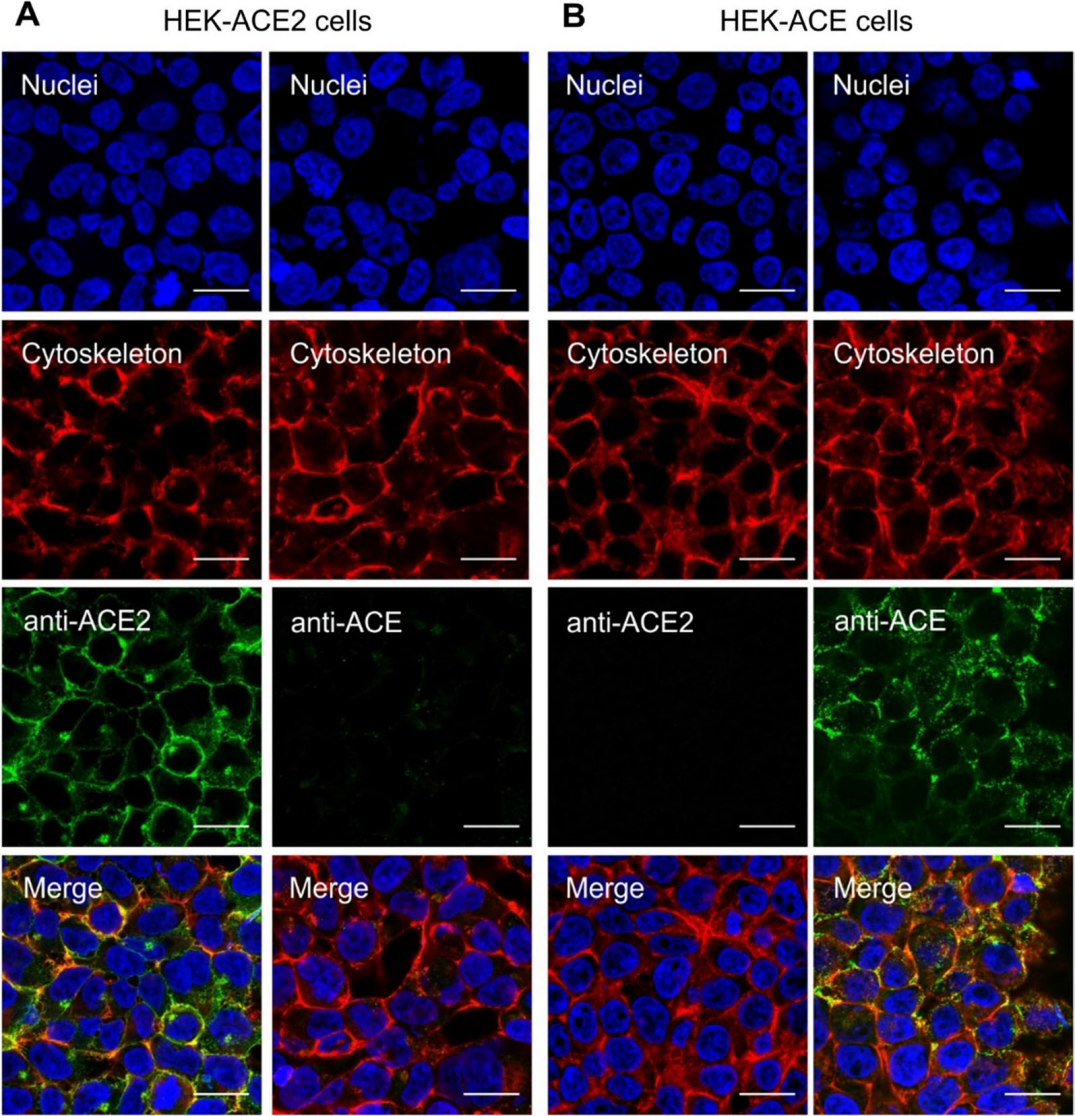
Immunofluorescence microscopy images of **A** HEK-ACE2 and **B** HEK-ACE cells. Nuclei were stained with DAPI (blue); F-actin of the cytoskeleton was stained with fluorescent phalloidin (red); ACE2 and ACE were stained with a fluorophore-conjugated secondary antibody (green) after incubation with anti-ACE2 and anti-ACE antibodies; The merged images show the overlay of all stainings. The scale bar corresponds to 20 μm

Expression of ACE2 or ACE on the respective HEK cell lines was verified using immunofluorescence microscopy. HEK-ACE and HEK-ACE2 cells grown on coverslips were fixed using paraformaldehyde solution (4%). After rinsing with PBS, the cells were incubated with the blocking solution [5% bovine serum albumin (BSA)] containing Triton X-100 in PBS. After removal of the blocking solution, mouse anti-ACE2 antibody (1:200 diluted; Santa Cruz Biotechnology, Inc. sc-390851) and mouse anti-ACE (1:50 diluted; Santa Cruz Biotechnology, Inc. sc-23908) in 1% BSA were added, followed by incubation at 4 °C overnight. The cells were rinsed with PBS before incubation with equine anti-mouse secondary antibody conjugated with AlexaFluor^®^ 488 (1:500 dilution in 1% BSA; abcam ab150105) for 1 h protected from light. The samples were stained with DyLight™ 554 phalloidin and 4′,6-diamidino-2-phenylindole (DAPI) to visualize F-actin of the cytoskeleton and nuclei, respectively, followed by rinsing with PBS and mounting onto glass slides using ProLong Gold Antifade mounting agent. Fluorescence images were acquired using a confocal microscope (Leica Stellaris 5). The images were analyzed using the ImageJ software version 1.52d. ACE2 and ACE were visualized on the HEK-ACE2 and HEK-ACE cell line, respectively (Fig. 2). The expression of ACE2 and ACE on the respective HEK cell lines was also confirmed by Western blot (Additional file 1).

### Cell uptake of the radiopeptides

The uptake and internalization of [^67^Ga]Ga-DOTA-DX600, [^67^Ga]Ga-NODAGA-DX600, [^67^Ga]Ga-HBED-CC-DX600 and [^67^Ga]Ga-DOTA-BPP9a were determined using HEK-ACE2 and HEK-ACE cells. The cells were seeded in poly-D-lysine-coated 12-well plates allowing cell adhesion and growth overnight. After rinsing the cells with PBS, they were incubated with the respective radiopeptide (25 µL, 1.9 pmol, 38 kBq). In some cell samples, the radiopeptides were co-incubated with excess of cyclo-DX600 (2 µM) or lisinopril (4 µM) to block ACE2 or ACE, respectively. After incubation of the cells for 1 h or 3 h, they were rinsed with PBS or acidic stripping buffer (glycine buffer with NaCl 0.9%, pH 2.8) to determine the total uptake and internalization of the radiopeptides, respectively. Cell samples were lysed using NaOH (1 M, 1 mL) before counting the activity in a *γ*-counter. The results were expressed as a percentage of total added activity and normalized to an average content of ~ 0.3 mg protein per well.

### ACE2- and ACE-binding affinity of the radiopeptides

In order to determine the *K*_D_ values of the DX600-based radiopeptides and [^67^Ga]Ga-DOTA-BPP9a, HEK-ACE2 and HEK-ACE cells were seeded in 48-well-plates allowing adhesion and growth overnight. The cell samples were incubated with increasing concentrations (1‒2000 nM) of the radiopeptides (5 MBq/nmol) on ice. Some of the cell samples were co-incubated with an excess of cyclo-DX600 or lisinopril (20 µM, 10 nmol) to block ACE2 or ACE, respectively, enabling the determination of non-specific binding (Additional file 1). After 1 h incubation, the cells were rinsed with PBS and lysed with NaOH (1 M, 600 µL) to count the activity in a *γ*-counter. The *K*_D_ values were determined by plotting specific binding (total binding minus unspecific binding) against the molar concentration of the added radiopeptide. A nonlinear regression analysis was performed using GraphPad Prism software (version 8.3.1).

### In vitro plasma stability of the radiopeptides

The ^67^Ga-labeled DX600-radiopeptides (20 MBq/nmol) were incubated in human and mouse plasma (10 MBq/200 µL), respectively, at 37 °C. Aliquots were taken at variable time points up to 24 h for analysis using reversed phase thin layer chromatography (TLC) with citrate buffer (0.1 M, pH 5.5) as a mobile phase. Images of the TLC plates were obtained using a storage phosphor system (Cyclone Plus, PerkinElmer) and quantified using the OptiQuant software (version 5.0, Bright Instrument Co Ltd., PerkinElmer™) (Supplementary Information).

### In vivo studies—mouse model

All applicable international, national, and/or institutional guidelines for the care and use of animals were followed. In particular, all animal experiments were carried out according to the guidelines of the Swiss Regulations for Animal Welfare. Preclinical studies have been ethically approved by the Cantonal Committee of Animal Experimentation and permitted by the responsible cantonal authorities (License N° 75743). Five-week-old female CD1/nude mice and female immunocompetent FVB mice were obtained from Charles River Laboratories (Sulzfeld, Germany) and fed with standard rodent chow ad libitum. CD1/nude were subcutaneously inoculated with HEK-ACE2 cells (6–8 × 10^6^ cells in 100 µL PBS) on the right shoulder and approximately one week later with HEK-ACE (4 × 10^6^ cells in 100 µL PBS) on the left shoulder. Imaging and biodistribution studies were performed 2–4 weeks later when the xenografts reached a size of 100–300 mm^3^.

### Biodistribution studies in xenografted mice

Biodistribution studies in xenograft-bearing mice were performed 2–4 weeks after cell inoculation. Mice were intravenously injected with [^67^Ga]Ga-DOTA-DX600, [^67^Ga]Ga-NODAGA-DX600, [^67^Ga]Ga-HBED-CC-DX600 or [^67^Ga]Ga-DOTA-BPP9a (3 MBq, 0.5 nmol, 100 µL) diluted in NaCl 0.9% containing 0.05% BSA. The mice were sacrificed 3 h after administration of the radiopeptides. Selected tissues and organs were collected, weighed and counted using a *γ*-counter. The results were listed as a percentage of the injected activity per gram of tissue mass (% IA/g), using counts of a defined volume of the original injection solution measured simultaneously, resulting in decay-corrected values. Statistical analysis was performed by applying a one‐way ANOVA test with a Tukey’s multiple comparisons post‐test (GraphPad Prism software, version 8.3.1).

### SPECT/CT imaging studies

SPECT/CT imaging was performed using a four-head, multiplexing, multipinhole small-animal SPECT camera (NanoSPECT/CT™, Mediso Medical Imaging Systems, Budapest, Hungary). Each head was outfitted with a tungsten-based aperture of nine 1.4 mm-diameter pinholes and a thickness of 10 mm. CT scans of 7.5 min duration were followed by SPECT scans of ~ 50 min performed 1 h, 3 h and 24 h after injection of the respective radiopeptide (10 MBq, 0.5 nmol, 100 μL), diluted in NaCl 0.9% containing 0.05% BSA. During the scan, mice were anesthetized with a mixture of isoflurane and oxygen. The images were acquired using Nucline software (version 1.02, Mediso Ltd., Budapest, Hungary). The real-time CT reconstruction used a cone-beam-filtered backprojection. The reconstruction of SPECT data was performed with the HiSPECT software (version 1.4.3049, Scivis GmbH, Göttingen, Germany) using *γ*-energies of 93.20 keV (± 10%), 184.60 keV (± 10%) and 300.00 keV (± 10%) for gallium-67. All images were prepared using the VivoQuant post-processing software (version 3.0, inviCRO Imaging Services and Software, Boston, US). A Gauss post-reconstruction filter (full width at half maximum, 1 mm) was applied, and the scale of activity for gallium-67 was set as indicated on the SPECT/CT images. In order to estimate the wash-out of the DX600-based radiopeptides from the HEK-ACE2 xenografts and kidneys over time, the absolute amount of activity was determined in these tissues using the VivoQuant quantification tool. The accumulated activity (non-decay-corrected value in MBq) in the xenografts and kidneys, respectively, obtained at the 1 h p.i. timepoint was set as 100%. The percentage of activity retained in these tissues was determined at 3 h and 24 h p.i. based on the absolute activity determined at these timepoints.

### In vivo stability of the radiopeptides

In order to investigate the stability of the radiopeptides, the ^67^Ga-labeled DX600 peptides (20 MBq/nmol) were tested in immunocompetent FVB mice (10 MBq in 100 μL per mouse). Before sacrificing, blood was collected from each mouse 1 h after injection of the radiopeptides. After centrifugation, the blood plasma was analyzed using reversed phase TLC with citrate buffer (0.1 M, pH 5.5) as a mobile phase. The kidneys were collected and processed to investigate the presence of intact radiopeptides and potential metabolites using the same TLC system. The analysis of the chromatograms was performed as described for the in vitro plasma stability (Additional file 1).

### In vitro autoradiography

Autoradiography studies were performed on frozen tissue sections of HEK-ACE2 and HEK-ACE xenografts. The sections were exposed to [^67^Ga]Ga-HBED-CC-DX600 (0.1–20 MBq/nmol; 0.5 MBq/mL) in Tris–HCl buffer (170 mM, pH 7.6, with 5 mM MgCl_2_) with 1% (w/v) BSA, with molar activities ranging from 0.1 to 20 MBq/nmol, for 60 min at room temperature. Cyclo-DX600 (10 µM) was added to block ACE2 binding. After incubation, the tissue sections were rinsed with buffer and MilliQ-water and air-dried. Images were obtained using a storage phosphor system (Cyclone Plus, PerkinElmer) and quantified using the OptiQuant software (version 5.0, Bright Instrument Co Ltd., PerkinElmer™). The specific binding to ACE2 and ACE was calculated by subtracting the signal intensity measured as digital light unit (DLU)/mm^2^ on the slides incubated with excess of cyclo-DX600 from the DLU/mm^2^ signal of the sections incubated with only the radiopeptide. The specific binding of the radiopeptide at various molar activities (0.1–20 MBq/nmol) was expressed as a percentage of the signal reached at 20 MBq/nmol which was set as 100%. The results are presented as average ± SD of three independent experiments, each performed with two replicates of HEK-ACE2 xenograft tissue and one replicate of HEK-ACE xenograft tissue.

## Results

### Orientation of the radiopeptide in the binding site of ACE2

**Fig. 3:**
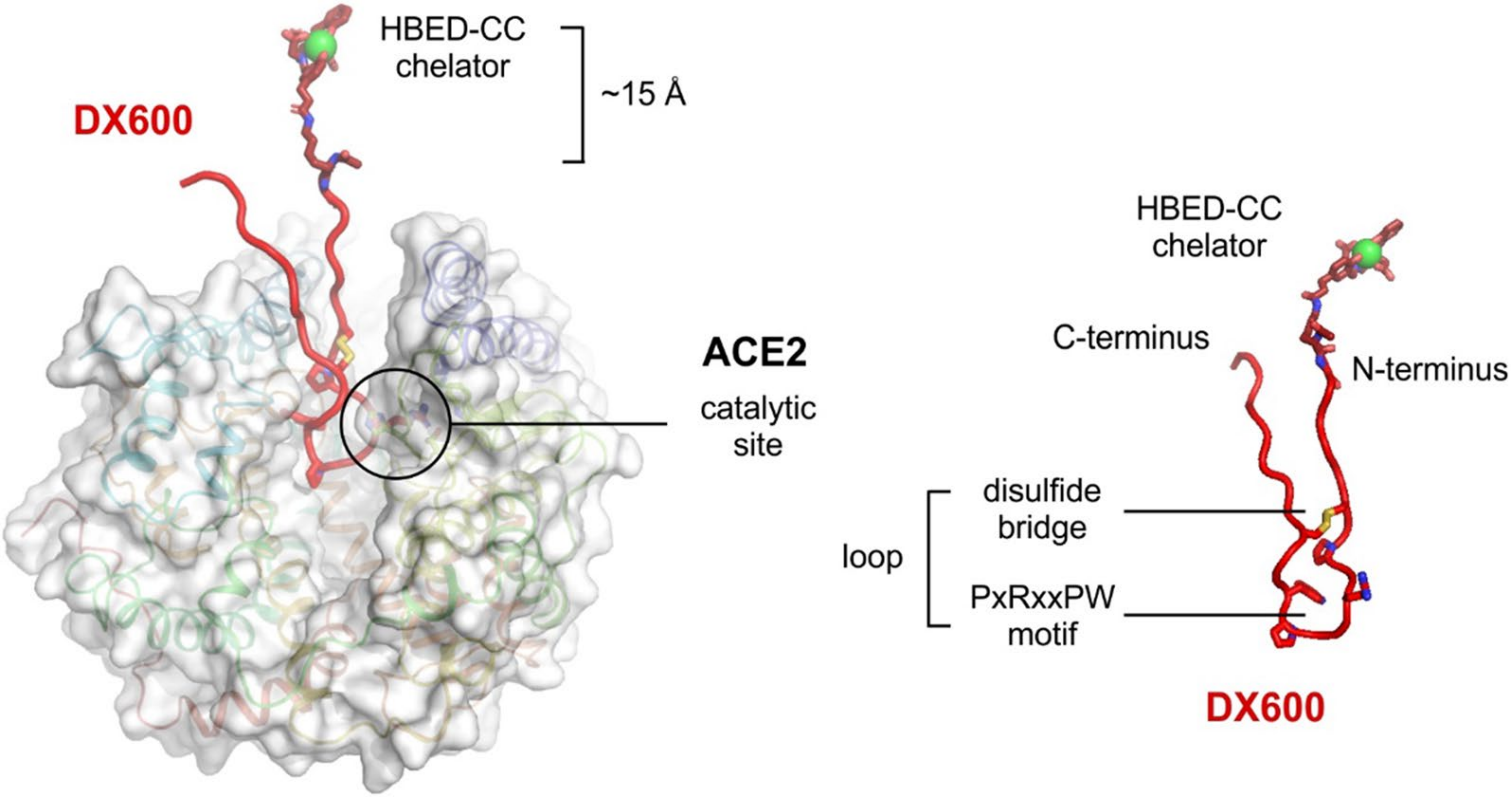
Molecular model of the cyclic DX600 peptide bound to ACE2 (left). The peptide is shown as a red cartoon and the protein is shown as cartoons and a translucent molecular surface. The central closed structured loop of DX600 results from a disulfide bridge between Cys6 and Cys17 (shown as sticks) and contains the PxRxxPW sequence motif (shown as sticks) that binds the catalytic site of ACE2. As a result, the N terminus of DX600 is solvent-exposed and accessible for modification with a chelator for radiometalation (in the figure, through a lysine-coupled HBED-CC shown as sticks enabling the coordination of gallium shown as a green sphere). An additional molecular model shows the Ga-HBED-CC-DX600 peptide structure separately (right)

The three-dimensional molecular model of DX600 bound to ACE2 suggests that both the C and N terminus of the cyclic peptide protrude from the binding groove that contains the catalytic site of ACE2 (Fig. 3). Thus, either end of the amino acid chain could be derivatized with a chelator without the need for a long spacer entity. In line with previous data of other groups [26, 27], the N terminus was selected as a more convenient site for modification of DX600. Our model shows that chelators coupled through a lysine would be around 15 Å away from the protein surface and, thus, not interfere with the binding of the peptide (in Fig. 3, the gallium-bound HBED-CC chelator is shown as an example). Based on this model, we designed DX600-based peptides conjugated to three different chelating agents: DOTA-DX600, NODAGA-DX600 and HBED-CC-DX600.

### Labeling efficiency of the DX600-based peptides using gallium-67

The ^67^Ga-labeling of DX600-based peptides and DOTA-BPP9a yielded radiopeptides with high radiochemical purities (> 98%) at a molar activity up to 20 MBq/nmol, the highest molar activity used in these studies (Additional file 1: Fig. S3). The DX600-based radiopeptides were entirely stable (≥ 99% intact product) in saline over a 24 h-time period. Partial radiolysis (~ 11% degradation products) was seen in the case of [^67^Ga]Ga-DOTA-BPP9a after 24 h incubation; however, it was sufficiently stable (> 99%) over the first 3 h after preparation during which the experiments were performed (Additional file 1: Table S3).

**Fig. 4:**
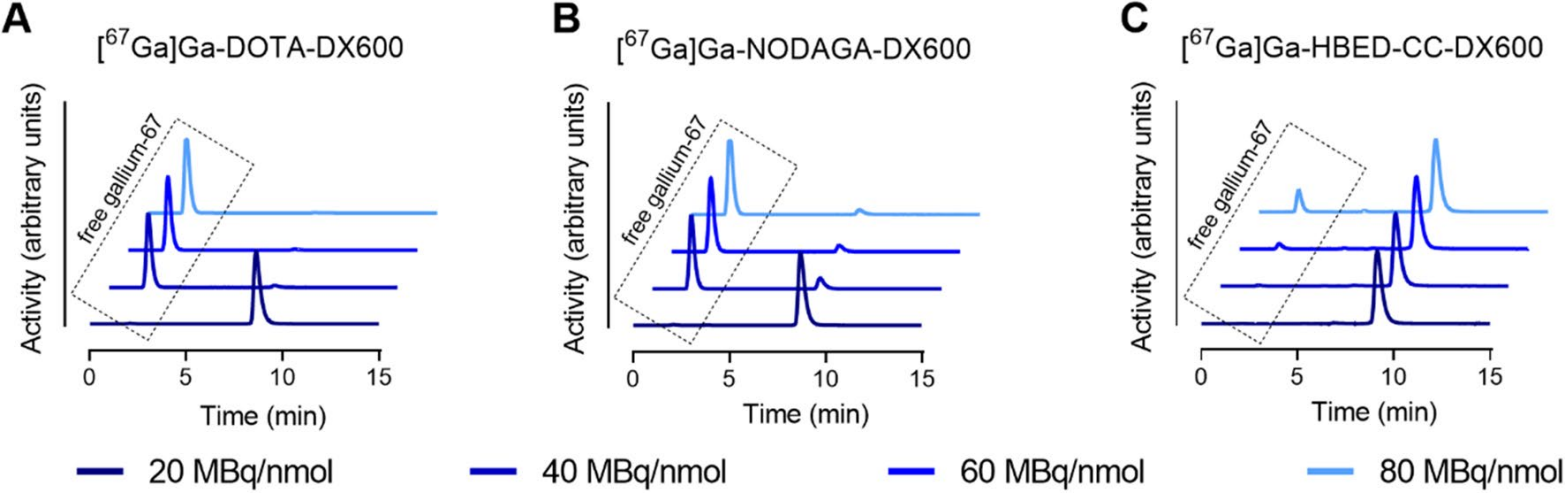
Representative HPLC chromatograms of the radiopeptides prepared at variable molar activities (20–80 MBq/nmol). **A** [^67^Ga]Ga-DOTA-DX600; **B** [^67^Ga]Ga-NODAGA-DX600 and **C** [^67^Ga]Ga-HBED-CC-DX600. The retention time (*t*_R_) of un-coordinated (“free”) gallium-67 was ~ 2.5 min; the *t*_R_ of radiopeptides was ~ 9.0 min

The labeling efficiency of the DX600-based peptides was assessed using the highest possible molar activity that would still allow the preparation of the DX600-based radiopeptide at a radiochemical purity of > 95% (Fig. 4). The data showed that ^67^Ga-labeling of HBED-CC-DX600 was feasible at a threefold higher molar activity (60 MBq/nmol; radiochemical purity: 96%) than NODAGA-DX600 (40 MBq/nmol; 16% and 20 MBq/nmol; 99%), which was still slightly favorable over the use of DOTA-DX600 (40 MBq/nmol; 3.0% and 20 MBq/nmol; 99%) (Additional file 1: Table S4). These experimental findings indicated an advantage of using HBED-CC over the macrocyclic chelators NODAGA and DOTA for the preparation of the radiopeptide at high molar activities.

### Distribution coefficient and blood plasma protein-binding properties of the radiopeptides

The distribution coefficients, indicated as logD values, of [^67^Ga]Ga-DOTA-DX600 (− 3.06 ± 0.06) and [^67^Ga]Ga-NODAGA-DX600 (− 3.05 ± 0.10) were equal. [^67^Ga]Ga-HBED-CC-DX600 showed a slightly lower logD value of − 3.35 ± 0.25, indicating an enhanced polarity of this radiopeptide. The structurally different [^67^Ga]Ga-DOTA-BPP9a had a lower logD value (− 4.60 ± 0.14).

The blood plasma protein-binding capacity was in the same range for all three DX600-based radiopeptides irrespective of whether human (78–83%) or mouse blood plasma (78–82%) was used. On the other hand, a much lower fraction of [^67^Ga]Ga-DOTA-BPP9a was found to bind to human (~ 49%) and mouse blood plasma proteins (~ 53%) (Additional file 1: Fig. S4).

### ACE2-selective binding of the DX600-based radiopeptides

**Fig. 5:**
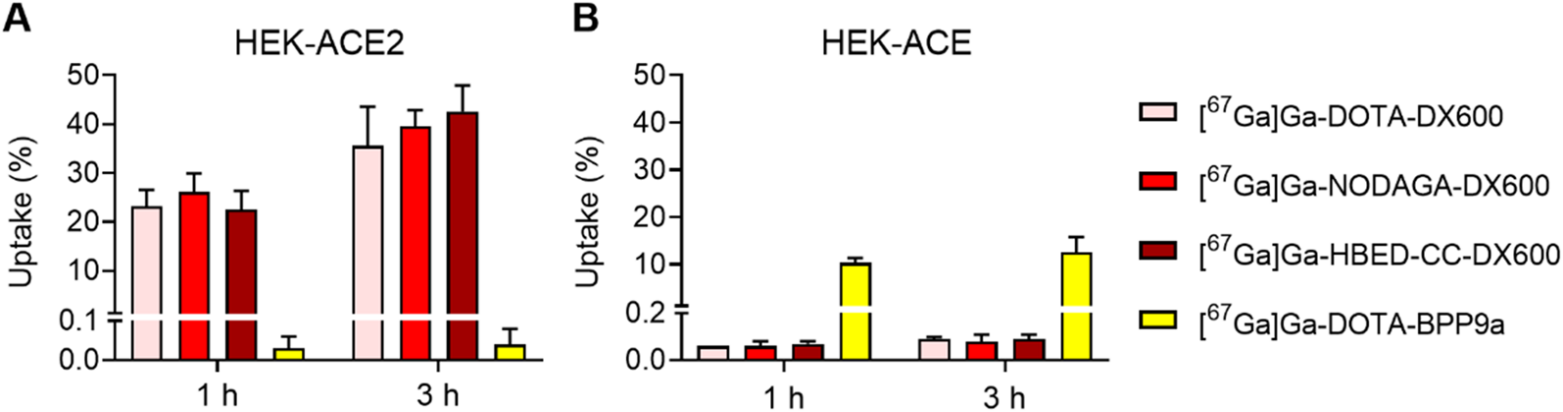
Uptake of the radiopeptides in **A** HEK-ACE2 and **B** HEK-ACE cells

Cell experiments were performed with HEK cells that expressed either ACE2 or ACE as demonstrated by immunofluorescence microscopy and Western blot (Fig. 2; Additional file 1: Fig. S5). The uptake of [^67^Ga]Ga-DOTA-DX600, [^67^Ga]Ga-NODAGA-DX600 and [^67^Ga]Ga-HBED-CC-DX600 in HEK-ACE2 cells was 36 ± 8%, 40 ± 3% and 43 ± 5%, respectively, after a 3 h-incubation period (Fig. 5A). The internalized fractions following the radiopeptides' binding were relatively low (6.1 ± 1.8%, 8.2 ± 0.3%, and 8.7 ± 0.5%, respectively). Studies performed by incubation of the radiopeptides in the presence of an excess of cyclo-DX600 to block ACE2 revealed that only a small fraction of [^67^Ga]Ga-DOTA-DX600 (2.4 ± 2.0%), [^67^Ga]Ga-NODAGA-DX600 (3.1 ± 1.1%), and [^67^Ga]Ga-HBED-CC-DX600 (1.0 ± 0.6%) was unspecifically bound to the cells (Additional file 1: Fig. S6). Determination of the ACE2-binding affinity of the ^67^Ga-labeled DOTA-DX600, NODAGA-DX600 and HBED-CC-DX600 revealed *K*_*D*_ values of 98 ± 10 nM, 83 ± 19 nM and 113 ± 17 nM, respectively. The selectivity of the radiopeptides toward ACE2 was demonstrated by the negligible uptake (< 0.1%) of the DX600-based radiopeptides in HEK-ACE cells (Fig. 5B). On the other hand, [^67^Ga]Ga-DOTA-BPP9a showed specific uptake in ACE-expressing HEK cells (16 ± 2%) after a 3 h-incubation period (Fig. 5B), but the binding affinity was only moderate (693 ± 354 nM).

### Plasma stability of the radiopeptides

In mouse blood plasma, [^67^Ga]Ga-DOTA-DX600 was intact over the first hour, however, after 3 h and 24 h incubation at 37 °C only 95 ± 3% and 20 ± 13% intact radiopeptide was detected, respectively. A similar trend was observed for [^67^Ga]Ga-NODAGA-DX600 and [^67^Ga]Ga-HBED-CC-DX600 with 97 ± 1% and 87 ± 2% intact radiopeptide after 3 h incubation, respectively, and 36 ± 7% and 29 ± 1% intact radiopeptide after 24 h incubation, respectively. A release of gallium-67 was observed only in the case of [^67^Ga]Ga-DOTA-DX600 (~ 4% after 3 h and > 50% after 24 h), but not for the other two DX600-based radiopeptides.

In human blood plasma, [^67^Ga]Ga-NODAGA-DX600 and [^67^Ga]Ga-HBED-CC-DX600 were entirely stable (> 99% intact radiopeptide) over the whole incubation period of 24 h. Only in the case of [^67^Ga]Ga-DOTA-DX600, a substantial amount of released gallium-67 (> 30%) was detected after 24 h incubation. None of the radiopeptides showed any signs of radiometabolite formation over the whole time of investigation in human blood plasma (Additional file 1: Fig. S7).

### Biodistribution and imaging studies in HEK-ACE2/HEK-ACE xenografted mice

Biodistribution studies were performed 3 h after injection of the radiopeptides into HEK-ACE2/HEK-ACE-xenograft-bearing mice (Additional file 1: Table S5). At this timepoint, the activity was entirely cleared from the blood pool (< 0.1% IA/g). Considerable uptake of [^67^Ga]Ga-DOTA-DX600, [^67^Ga]Ga-NODAGA-DX600 and [^67^Ga]Ga-HBED-CC-DX600 was observed in HEK-ACE2 xenografts (10.7 ± 0.4% IA/g, 15.5 ± 2.2% IA/g, and 12.3 ± 1.3% IA/g, respectively, *p* > 0.05), but only negligible accumulation of these peptides was seen in the HEK-ACE xenografts (< 0.5% IA/g, 3 h p.i.). In non-targeted organs and tissues only background activity was observed (< 0.5% IA/g) with the only exception of the kidneys. Renal retention of [^67^Ga]Ga-DOTA-DX600 and [^67^Ga]Ga-NODAGA-DX600 was much higher (24.0 ± 0.8% IA/g and 23.9 ± 4.5% IA/g, 3 h p.i.) than that of [^67^Ga]Ga-HBED-CC-DX600 (7.2 ± 2.2% IA/g, *p* < 0.05). As a result, the HEK-ACE2 xenograft-to-kidney ratio of [^67^Ga]Ga-HBED-CC-DX600 (1.77 ± 0.34; *p* < 0.05) was more than twofold higher than that of [^67^Ga]Ga-NODAGA-DX600 (0.67 ± 0.16) and more than threefold higher than that of [^67^Ga]Ga-DOTA-DX600 (0.45 ± 0.02).

**Fig. 6:**
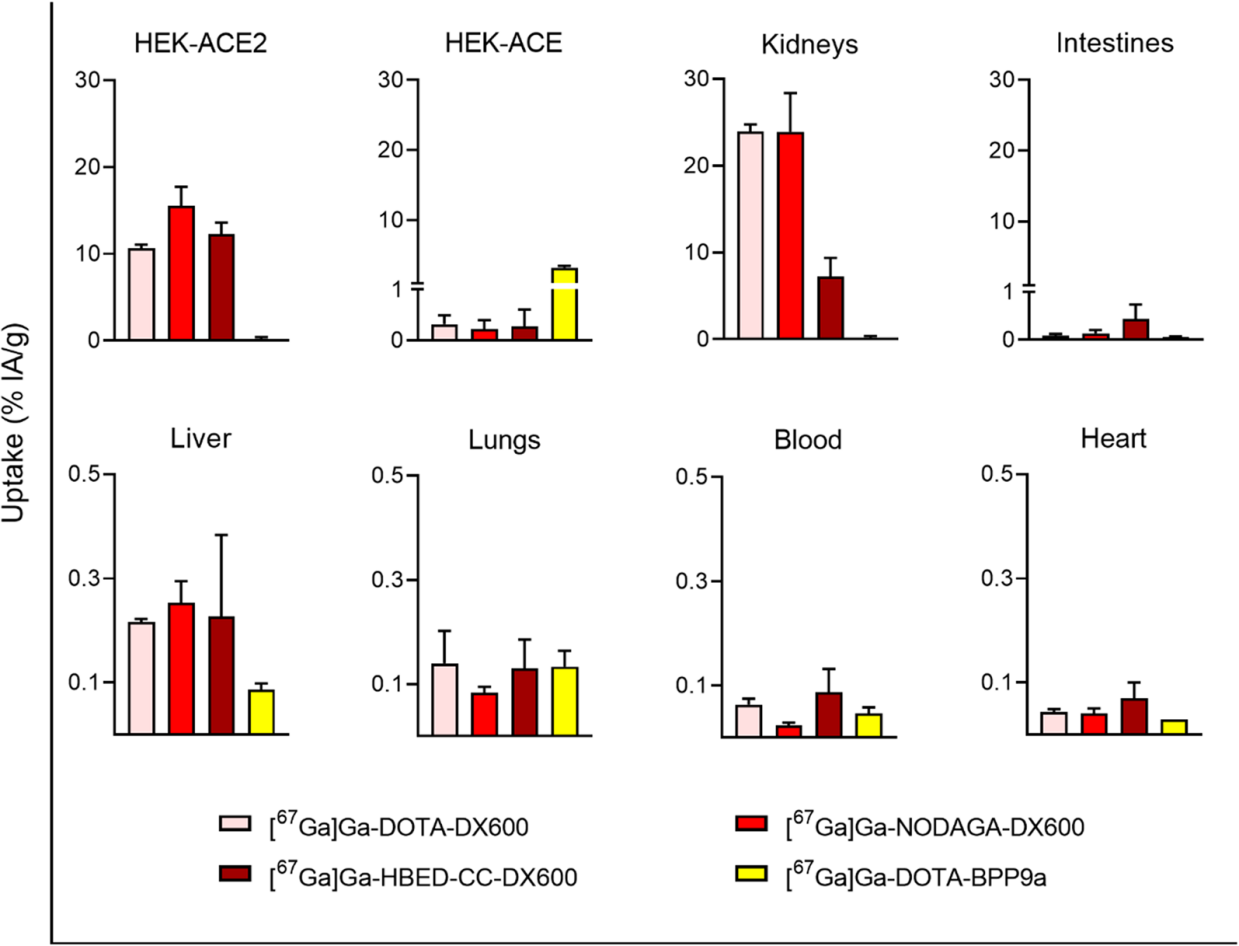
Biodistribution data of [^67^Ga]Ga-DOTA-DX600, [^67^Ga]Ga-NODAGA-DX600, [^67^Ga]Ga-HBED-CC-DX600 and [^67^Ga]Ga-DOTA-BPP9a in selected organs obtained at 3 h after injection into HEK-ACE2/ACE xenograft-bearing mice (average ± SD, n = 3)

[^67^Ga]Ga-DOTA-BPP9a accumulated selectively in the HEK-ACE xenograft (3.17 ± 0.29% IA/g), while negligible uptake was observed in the HEK-ACE2 xenograft (< 0.5% IA/g). This data confirmed the protein expression in this xenograft type and served for validation of the ACE2-selectivity profile of the DX600-based radiopeptides (Fig. 6).

### SPECT/CT imaging studies using HEK-ACE2/HEK-ACE-xenografted mice

**Fig. 7:**
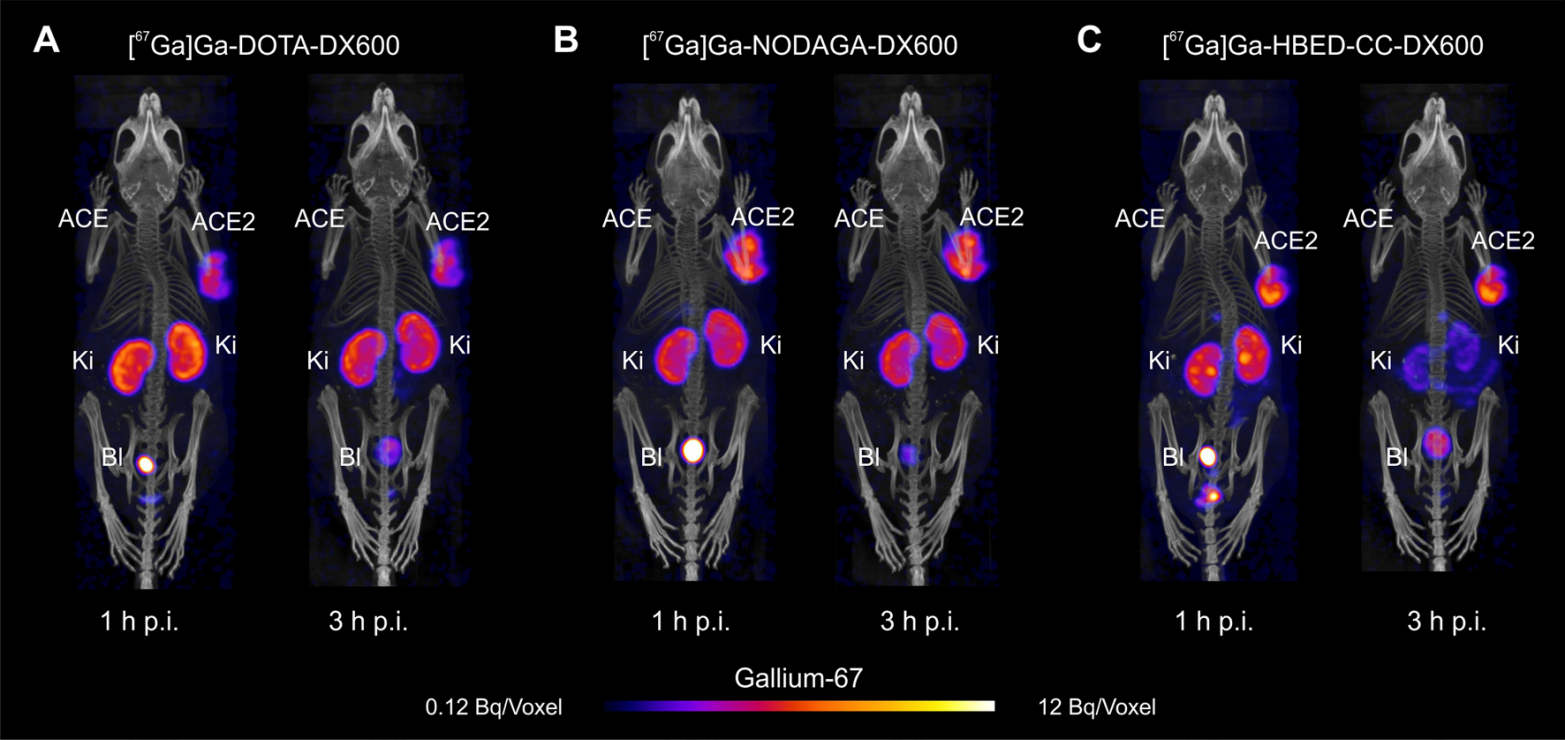
Representative SPECT/CT images of HEK-ACE2/ACE xenograft-bearing mice obtained 1 h and 3 h after injection of the radiopeptides. **A** [^67^Ga]Ga-DOTA-DX600; **B** [^67^Ga]Ga-NODAGA-DX600 and **C** [^67^Ga]Ga-HBED-CC-DX600

SPECT/CT imaging studies of mice conducted at 1 h and 3 h after injection of the DX600-based radiopeptides revealed the activity accumulation in ACE2-expressing xenografts but not in those that expressed ACE. The radiopeptides were well retained in the HEK-ACE2 xenografts over time showing still about 92–95% and about 47–58% of the activity in the xenografts after 3 h and 24 h, respectively, compared to the 1 h p.i.-timepoint. All three radiopeptides showed retention in the kidneys as a result of renal excretion. This was particularly prominent after injection of [^67^Ga]Ga-DOTA-DX600 and [^67^Ga]Ga-NODAGA-DX600 (Fig. 7A/B), for which ~ 85% of the measured activity at the 1 h p.i.-timepoint was retained after 3 h and still about 35–45% after 24 h. In the case of [^67^Ga]Ga-HBED-CC-DX600, kidney clearance was much faster (Fig. 7C), demonstrated by only 30% of the activity left in the kidneys after 3 h relative to the amount measured at the 1 h p.i.-timepoint. After 24 h, [^67^Ga]Ga-HBED-CC-DX600 was entirely excreted from the kidneys (< 1% of the activity measured at the 1 h p.i.-timepoint).

ACE-selective accumulation of activity was visible in the HEK-ACE xenograft after injection of [^67^Ga]Ga-DOTA-BPP9a (Additional file 1: Fig. S8). These data confirmed the feasibility of selectively targeting ACE2 in the presence of ACE using DX600-based radiopeptides and, on the other hand, targeting ACE in the presence of ACE2 when using the BPP9a-based radiopeptide.

### In vivo stability of the radiopeptides

Blood plasma samples, urine and kidneys were analyzed to determine the presence of radiometabolites after injection of the DX600-based radiopeptides (Additional file 1: Fig. S9). The analysis of blood plasma samples collected from mice at 1 h p.i. of the DX600-based radiopeptides revealed the formation of radiometabolites (3–13% of the total activity determined in the blood). Only in the case of [^67^Ga]Ga-DOTA-DX600, release of gallium-67 from the chelator was observed (3.5% of the total activity determined in the blood). In urine samples taken at the same timepoint, a major fraction (59–73%) of the measured activity was ascribed to radiometabolites irrespective of the injected radiopeptide. The analysis of the kidney homogenates revealed that a large fraction of the accumulated activity was due to radiometabolite formation after injection of [^67^Ga]Ga-DOTA-DX600 (~ 93%) and [^67^Ga]Ga-NODAGA-DX600 (~ 90%), respectively. This was, however, not the case in mice that received [^67^Ga]Ga-HBED-CC-DX600, in which almost all activity accumulated in the kidneys could be ascribed to the intact radiopeptide (> 92%), whereas only a small fraction was attributed to radiometabolites (~ 7.5%).

### In vitro autoradiography on HEK-ACE2 and HEK-ACE xenograft tissue

**Fig. 8:**
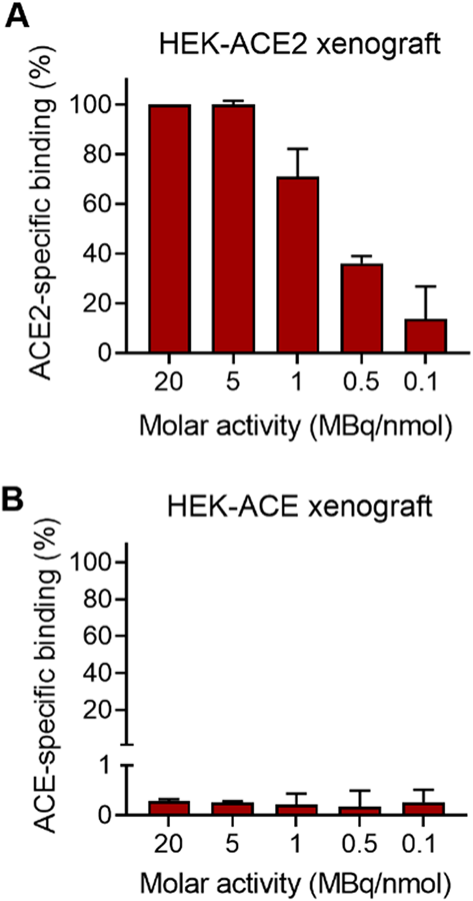
Quantification of autoradiograms obtained after incubation of **A** frozen HEK-ACE2 xenograft sections and **B** frozen HEK-ACE xenograft sections to [^67^Ga]Ga-HBED-CC-DX600. The signal intensity is presented as the percentage of the signal intensity obtained from the HEK-ACE2 xenograft section incubated with [^67^Ga]Ga-HBED-CC-DX600 labeled at the highest molar activity (20 MBq/nmol) which was set as 100%

The impact of the radioligand’s molar activity and, therewith, the fraction of labeled peptide relative to the total amount of used peptide, was demonstrated using the method of in vitro autoradiography. Frozen tissue sections of HEK-ACE2 and HEK-ACE xenografts were exposed to [^67^Ga]Ga-HBED-CC-DX600 prepared at variable molar activities. No significant differences in tissue binding were observed when using molar activities of 5 MBq/nmol or higher. Application of the radioligand prepared at lower molar activities (0.1–1 MBq/nmol) quantitatively affected the radiopeptide’s receptor binding due to saturation effects by unlabeled peptide. At a molar activity of 0.1 MBq/nmol, the binding was reduced to 14% of the value obtained with a molar activity of 5 MBq/nmol or higher (Fig. 8A). No specific binding of [^67^Ga]Ga-HBED-CC-DX600 was observed on HEK-ACE tissue sections, regardless of the employed molar activity of the radiopeptide (Fig. 8B). Representative autoradiograms of HEK-ACE2 and HEK-ACE xenografts are reported in Additional file 1: Fig. S10.

## Discussion

In order to develop a suitable ACE2-binding radiopeptide, the compatibility of conjugating a chelator to DX600 without affecting its ACE2-binding capability was assessed. Computational models based on experimental NMR and X-ray crystallography data [25] for DX600 and ACE2, respectively, suggested that a direct fusion to the chelator (i.e., without the need for a long linker) should be feasible at both the N or C terminus of the peptide without compromising its receptor-binding properties. With this information, three DX600-based peptides were designed using DOTA, NODAGA and HBED-CC as metal chelators, which were conjugated to the N terminus of the peptide.

The overall physicochemical properties of the resultant ^67^Ga-labeled peptides (logD values and stability) were not significantly affected by the type of chelator that was employed. In vitro experiments using HEK-ACE2 and HEK-ACE cells, validated by immunofluorescence imaging and Western blot, confirmed the anticipated ACE2-specific binding and uptake of the DX600-based radiopeptides whereas [^67^Ga]Ga-DOTA-BPP9a bound to ACE, but not to ACE2.

Biodistribution studies in HEK-ACE2/HEK-ACE xenografted mice demonstrated similar accumulation of activity in HEK-ACE2 xenografts at 3 h after injection of all three DX600-based radiopeptides while none of them accumulated in HEK-ACE xenografts. This is of high relevance as the selective visualization of ACE2 would be essential to investigate its expression and dynamics and enable a potential correlation with Covid-19 phenotype and disease severity.

In normal tissues and organs, negligible accumulation of the DX600-based radiopeptides was observed which can be likely attributed to the low affinity of DX600 to the murine ACE2 protein [36]. In the kidneys, we found, however high accumulation of activity after injection of [^67^Ga]Ga-DOTA-DX600 and [^67^Ga]Ga-NODAGA-DX600. This was in line with previously published data of other groups using similar radiopeptides with a DOTA or NODAGA chelator, respectively [27]. Importantly, [^67^Ga]Ga-HBED-CC-DX600 demonstrated a more favorable tissue distribution profile than the other two DX600-based radiopeptides with high activity accumulation in HEK-ACE2 xenografts but reduced retention in the kidneys. These findings were in agreement with SPECT/CT images which showed the highest signal-to-background ratio for [^67^Ga]Ga-HBED-CC-DX600. This radiopeptide clearly outperformed the other two and any previously-developed DX600-based radiopeptide [27]. The improved signal-to-background contrast will be essential for visualizing small sites of ACE2 expression in critical organs and tissues, such as the lungs and vasculature of Covid-19 patients.

An open question remained, however, why [^67^Ga]Ga-DOTA-DX600 and [^67^Ga]Ga-NODAGA-DX600 showed such high kidney retention although they shared the targeting scaffold with [^67^Ga]Ga-HBED-CC-DX600. The investigation of blood plasma samples 1 h after injection of each radiopeptide indicated the formation of radiometabolites which were present in urine samples as the predominant fraction of excreted activity. The analysis of the kidney tissue suggests that the radiometabolites of [^67^Ga]Ga-DOTA-DX600 and [^67^Ga]Ga-NODAGA-DX600 were retained in the kidneys over time leading to substantial activity accumulation. This was, however, much less the case for [^67^Ga]Ga-HBED-CC-DX600, for which only a very small fraction of radiometabolites were found in the kidneys. Over all, it appeared that [^67^Ga]Ga-HBED-CC-DX600 was more stable and the formed radiometabolites were obviously cleared effectively through kidneys.

Although the in vitro studies in blood plasma confirmed degradation of the radiopeptides over time, the fact that the metabolism was much less pronounced in human blood plasma raised hopes that the observed in vivo degradation would possibly be less dominant in humans. Finally, it has to be mentioned that DOTA is not an ideal chelator for coordination of gallium-67 due to observed release of the radiometal over time as shown for [^67^Ga]Ga-DOTA-DX600 and previously reported in the literature [37].

The most favorable features of [^67^Ga]Ga-HBED-CC-DX600 also included the more efficient and specific gallium complexation by HBED-CC as compared to that of the DOTA- and NODAGA-derivatized peptides. This can be ascribed to the fact that HBED-CC favors the coordination of gallium (Ga^3+^: logK: 38) over zinc (Zn^2+^: logK: 18) that is likely present in solution as a result of the decay of gallium radioisotopes, due to poor separation from the target material or as an environmental impurity [38]. In contrast, DOTA effectively coordinates also zinc [39] which would compete with gallium and prevent radiolabeling at high molar activity. In the present study, the use of radiopeptides prepared with high molar activity was not crucial to perform experiments with HEK-ACE2 cells/xenografts that express the receptor at high levels. It will be essential, however, that the non-labeled peptide fraction would be as small as possible in order to enable the visualization of the low (patho)physiological ACE2 expression levels in diverse tissues since saturation effects due to the presence of non-labeled peptides would reduce the signal-to-background contrast. The data resulting from autoradiographies performed with [^67^Ga]Ga-HBED-CC-DX600 prepared at variable molar activities indicated that application of the radiopeptide at high molar activity is advantageous to optimize the amount of radiopeptide which bound to frozen xenograft sections.

Finally, since the signal-to-background contrast increases over time in vivo, it is likely that nuclear imaging at later time points would be preferred for detecting small sites of ACE2 expression. In this regard, the use of the short-lived gallium-68 (*T*_1/2_ = 68 min) may not be ideal, however; gallium-67 (*T*_1/2_ = 3.26 d) would enable SPECT imaging at later time points. This would require, however, the commercial availability of ^67^Ga-chloride at a high quality that allowed preparation of these radiopeptides under Good Manufacturing Procedure (GMP) conditions.

## Conclusion

Our data identified [^67^Ga]Ga-HBED-CC-DX600 as a promising nuclear imaging agent to investigate the in vivo expression profile and dynamics of ACE2 for a better understanding of Covid-19 pathology. The biodistribution of [^67^Ga]Ga-HBED-CC-DX600 outperformed that of other DX600-based radiopeptides with DOTA or NODAGA chelators. Importantly, the HBED-CC chelator enabled ^67^Ga-labeling at high molar activity, which would be essential to obtain images with high signal-to-background contrast to detect (patho)physiological ACE2 expression levels in patients.

## Supplementary Information

**Additional file 1:** More detailed information about methods and results of the synthesis and preclinical evaluation of the radiopeptides.

## Data Availability

All preclinical data analyzed during this study are included in this published article and in supplementary Information. Additional information or more detailed data are available from the corresponding author on reasonable request.
