# Supplementary material for: A tool for nuclear imaging of the SARS-CoV-2 entry receptor: molecular model and preclinical development of ACE2-selective radiopeptides
Source: EJNMMI Res. 2023 Apr 19;13:32. doi: 10.1186/s13550-023-00979-2 (PMC10113987; doi:10.1186/s13550-023-00979-2)
Supplement: Supplementary file 1 — Additional file 1: More detailed information about methods and results of the synthesis and preclinical evaluation of the radiopeptides. [file 13550_2023_979_MOESM1_ESM.docx]

Supplementary Material

**A tool for nuclear imaging of the SARS-CoV-2 entry receptor: Molecular model and preclinical development of ACE2-selective radiopeptides**

Darja Beyer^1†^, Christian Vaccarin^1†^, Xavier Deupi^2,3,4^, Ana Katrina Mapanao^1^, Susan Cohrs^1^, Fan Sozzi-Guo^1^, Pascal V. Grundler^1^, Nicholas P. van der Meulen^1,5^, Jinling Wang^6^, Matthias Tanriver^6^, Jeffrey W. Bode^6^, Roger Schibli^1,7^, Cristina Müller^1, 7^*

*1. Center for Radiopharmaceutical Sciences ETH-PSI, Paul Scherrer Institute, 5232 Villigen-PSI, Switzerland*

*2. Condensed Matter Theory Group, Division of Scientific Computing, Theory, and Data, Paul Scherrer Institute, 5232 Villigen-PSI, Switzerland*

*3. Laboratory of Biomolecular Research, Paul Scherrer Institute, 5232 Villigen-PSI, Switzerland*

*4. Swiss Institute of Bioinformatics (SIB), Lausanne, Switzerland*

*5. Laboratory of Radiochemistry, Paul Scherrer Institute, 5232 Villigen-PSI, Switzerland*

*6. Institute of Organic Chemistry, Department of Chemistry and Applied Biosciences, ETH Zurich, 8093 Zurich, Switzerland*

*7. Institute of Pharmaceutical Sciences, Department of Chemistry and Applied Biosciences, ETH Zurich, 8093 Zurich, Switzerland*

† equally contributed

*Correspondence to:

PD Dr. Cristina Müller

Center for Radiopharmaceutical Sciences ETH-PSI

Paul Scherrer Institute

5232 Villigen-PSI

Switzerland

e-mail: cristina.mueller@psi.ch

phone: +41 56 310 44 54; fax: +41 56 310 28 49

**1. Quality control of DX600-based peptides**

**Purpose:** The DX600-based peptides were obtained as a custom synthesis by piCHEM (Research & Development GmbH, Raaba-Grambach, Austria). The identity and purity of the peptides were confirmed before using them for the studies reported herein.

**Methods:** The purity of the DX600-based peptides was determined by analytical HPLC using a Merck-Hitachi LaChrom HPLC system equipped with a D-7000 interface, L-7200 autosampler, L-7400 UV detector, L-7100 pump and a reversed phase C18 column (Sunfire^TM^, 5 μm, 4.6 x 150 mm, Waters, Milford, MA, U.S.A.) at a wavelength of λ = 254 nm. The peptides were eluted using a linear gradient of Milli-Q water containing 0.1% trifluoroacetic acid (TFA) (95%–20%) and acetonitrile (5%–80%) over 15 min at a flow rate of 1.0 mL/min. The chemical identity of the peptides was assessed by high-resolution MALDI-TOF-MS (Bruker UltraFlex II; Billarica, MA, U.S.A.).

**Results:** The cyclic DX600 peptides, functionalized with an N-α-acetyl-lysine residue at the N terminus, were derivatized with a DOTA, NODAGA or HBED-CC chelator on the γ-amino group present on the sidechain (Table S1) to yield the desired DOTA-DX600, NODAGA-DX600 and HBED-CC-DX600, respectively. Moreover, the unmodified DX600 peptide, referred to as cyclo-DX600, was used for blocking experiments.

**Table S1** Amino acidic sequence and chelator functionalization of the DX600-based peptides.

| **Peptide name** | **Peptide sequence** |
| --- | --- |
| DOTA-DX600 | Acetyl-Lys(DOTA)-Gly-Asp-Tyr-Ser-His-Cys-Ser-Pro-Leu-Arg-Tyr-Tyr-Pro-Trp-Trp-Lys-Cys-Thr-Tyr-Pro-Asp-Pro-Glu-Gly-Gly-Gly-NH_2_ |
| NODAGA-DX600 | Acetyl-Lys(NODAGA)-Gly-Asp-Tyr-Ser-His-Cys-Ser-Pro-Leu-Arg-Tyr-Tyr-Pro-Trp-Trp-Lys-Cys-Thr-Tyr-Pro-Asp-Pro-Glu-Gly-Gly-Gly-NH_2_ |
| HBED-CC-DX600 | Acetyl-Lys(HBED-CC)-Gly-Asp-Tyr-Ser-His-Cys-Ser-Pro-Leu-Arg-Tyr-Tyr-Pro-Trp-Trp-Lys-Cys-Thr-Tyr-Pro-Asp-Pro-Glu-Gly-Gly-Gly-NH_2_ |
| Cyclo-DX600 | Acetyl-Gly-Asp-Tyr-Ser-His-Cys-Ser-Pro-Leu-Arg-Tyr-Tyr-Pro-Trp-Trp-Lys-Cys-Thr-Tyr-Pro-Asp-Pro-Glu-Gly-Gly-Gly-NH_2_ |

Cysteine residues involved in disulfide bridges are highlighted in red. DOTA = 2-(4,7,10-tris(carboxymethyl)-1,4,7,10-tetrazacyclododec-1-yl)acetic acid; NODAGA = 2,2′-(7-(4-((2-aminoethyl)amino)-1-carboxy-4-oxobutyl)-1,4,7-triazonane-1,4-diyl)diacetic acid, HBED-CC = 3-(3-((2-((5-(2-carboxyethyl)-2-hydroxyphenyl)methyl-(carboxymethyl)amino)ethyl-(carboxymethyl)amino)methyl)-4-hydroxyphenyl)propanoic acid.

The HPLC retention times were virtually identical for the DOTA-DX600 (t_R_= 9.16 min) and the NODAGA-DX600 (t_R_= 9.16 min), but slightly enhanced for the HBED-CC-DX600 (t_R_= 9.35 min). The measured chemical purity was >99% for all three DX600-based peptides (Fig. S1). The measured HRMS spectra correlated well with the calculated values (Table S2).


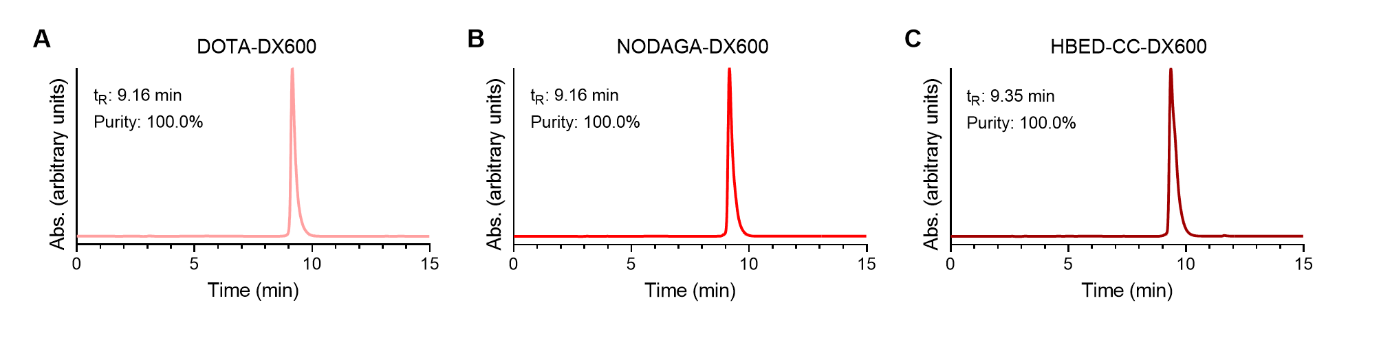


**Fig. S1** Representative UV-HPLC chromatograms obtained for **A** DOTA-DX600, **B** NODAGA-DX600 and **C** HBED-CC-DX600. The retention times (t_R_) and chemical purities (in %) are indicated in the figure.

**Table S2** HRMS (MALDI-TOF-MS) for the unlabeled ligands.

| Compound | Elemental composition | m/z (calcd.)^1^ | m/z (found)^2^ |
| --- | --- | --- | --- |
| DOTA-DX600 | C_163_H_223_N_41_O_48_S_2_ | 3588.5817 | 3588.4876 |
| NODAGA-DX600 | C_162_H_220_N_40_O_48_S_2_ | 3559.5552 | 3559.5920 |
| HBED-CC-DX600 | C_173_H_227_N_39_O_50_S_2_ | 3716.5967 | 3716.5019 |

^1^ theoretical m/z calculated as [M+H]^+^

^2^ measured m/z detected as [M+H]^+^

**2. Synthesis of DOTA-BPP9a**

**Purpose:** Angiotensin-converting enzyme (ACE) inhibitory peptides were previously isolated from the venom of *Bothrops jararaca* [1]. In this study, we adopted BPP9a to design an ACE-targeting radiopeptide.

**Methods:** The synthesis of the DOTA-modified BPP9a peptide was performed using solid-phase chemistry methods (Scheme S1). 2-Chlorotrityl chloride (2-CTC) resin (0.1 mmol) was weighed into a filter-containing 5 mL syringe and swelled in anhydrous dichloromethane (DCM) for 45 min. Fmoc-Pro-OH (0.12 mmol, 1.2 equiv) was dissolved in dry DCM in the presence of diisopropylethylamine (DIPEA, 0.8 mmol, 8.0 equiv), added to the resin, and stirred overnight (step **a**). Potentially unreacted 2-CTC resin was capped with a solution of DCM, methanol, and DIPEA (17:2:1, v/v/v) for 30 min. After conditioning in DMF, the Fmoc protecting group was removed by shaking the resin-immobilized compound in a mixture of DMF and piperidine in a ratio of 7:3 (v/v) twice for 5 min (step **b**). Fmoc-Pro-OH (0.4 mmol, 4.0 equiv), activated for 2 min with O-(benzotriazol-1-yl)-N,N,N′,N’-tetramethyluronium-hexafluorophosphate (HBTU, 0.396 mmol, 3.96 equiv) in the presence of DIPEA (0.8 mmol, 8.0 equiv) in dry DMF, was added to the resin and agitated for 45 min (step **c**). At this point, steps **b** and **c** were repeated with the appropriate Fmoc-protected amino acid derivatives [sequentially: Fmoc-Pro-OH, Fmoc-Ile-OH, Fmoc-Gln(Trt)-OH, Fmoc-Pro-OH, Fmoc-Arg(Pbf)-OH, Fmoc-Pro-OH, Fmoc-Trp(Boc)-OH, Fmoc-Glu(O^t^Bu)-OH, Fmoc-6-Ahx-OH, Fmoc-6-Ahx-OH], resulting in the desired peptide scaffold. Due to the lower reactivity of the secondary amino group in the proline residue, amino acids to be conjugated to a Pro residue (^iv^Trp, ^vi^Arg, ^ix^Ile, ^x^Pro) were coupled twice before proceeding with the Fmoc removal. For the final conjugation, the free carboxylic function of the DOTA-tri(^t^Bu) ester (0.2 mmol, 2.0 equiv) was activated for 2 min using (1-[Bis(dimethylamino)methylene]-1H-1,2,3-triazolo[4,5-b]pyridinium 3-oxide hexafluorophosphate (HATU, 0.198 mmol, 1.98 equiv) in the presence of DIPEA (0.4 mmol, 4.0 equiv) in dry DMF. This reaction mixture was added to the resin-immobilized compound and agitated for 45 min (step **d**). The resultant resin-immobilized intermediate was washed with DMF, DCM, and diethyl ether and dried under reduced pressure. Cleavage of the resultant compound from the resin and simultaneous removal of the acid-labile protecting groups was performed by exposing the resin-immobilized compound to a TFA solution containing 2.5% Milli-Q water and 2.5% triisopropylsilane (v/v/v) for 2 h (step **e**). TFA was removed by N_2_ stripping. The crude peptide was dissolved in a mixture of acetonitrile and Milli-Q water (50% v/v) and subsequently purified by semipreparative HPLC using a Merck-Hitachi LaChrom HPLC system equipped with a D-7000 interface, L-7200 autosampler, L-7400 UV detector, L-7100 pump and a reversed-phase C18 column (C18, Sunfire^TM^, 5 μm, 10×150 mm, Waters, Milford, MA, U.S.A.).

**Scheme S1** Solid-phase peptide synthesis of DOTA-BPP9a.

^1^ Sequence of coupled amino acids: Fmoc-Pro-OH, Fmoc-Ile-OH, Fmoc-Gln(Trt)-OH, Fmoc-Pro-OH, Fmoc-Arg(Pbf)-OH, Fmoc-Pro-OH, Fmoc-Trp(Boc)-OH, Fmoc-Glu(O^t^Bu)-OH, Fmoc-6-Ahx-OH, Fmoc-6-Ahx-OH.

The product was eluted using a linear gradient of Milli-Q water containing 0.1% TFA (95–50 %) and acetonitrile (5–50%) over 15 min at a flow rate of 2.0 mL/min. The fractions containing the pure product were detected by their absorbance (Abs) at λ = 254 nm, collected in a round-bottom flask, frozen in liquid nitrogen and lyophilized for ≥24 h. The chemical purity of the final DOTA-BPP9a was determined by UV-HPLC analysis (Sunfire^TM^, 5 μm, 4.6×150 mm, Waters, Milford, MA, U.S.A., λ = 254 nm) with a linear gradient of Milli-Q water containing 0.1% TFA (95–20%) and acetonitrile (5–80%) over 15 min at a flow rate of 1.0 mL/min.

**Results:** The desired peptide conjugate DOTA-BPP9a (DOTA-Ahx-Axh-Glu-Trp-Pro-Arg-Pro-Gln-Ile-Pro-Pro-OH) was obtained as a white powder (33.6 mg, 19.2 µmol, 19.2% yield) with a chemical purity of 99% (Fig. S2). Of note, the proline-rich sequence of DOTA-BPP9a resulted in metastable conformers that led to an asymmetrical elution profile during the HPLC analysis. This phenomenon was previously described for this class of compounds [2]. The chemical identity of the compound was confirmed by MALDI-TOF-MS analysis. HRMS (MALDI-TOF-MS) for C_81_H_126_N_20_O_22_ [M+H]^+^: calcd.: 1731.9429; found: 1731.9446.


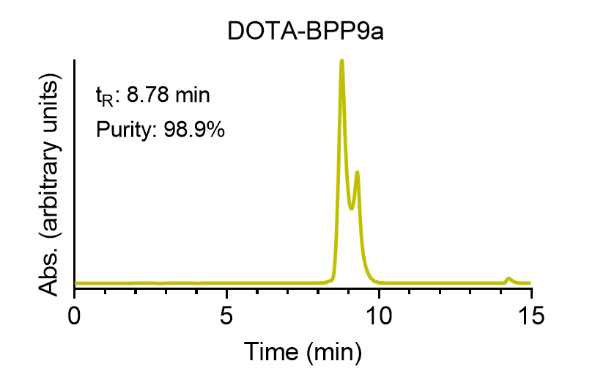


**Fig. S2** Representative UV-HPLC chromatogram for DOTA-BPP9a. The retention time (t_R_) and chemical purity (in %) are indicated in the figure.

**3. Preparation of ^67^Ga-labeled DX600 peptides**

**Purpose:** The DX600- and BPP9a-based peptides were labeled with gallium-67 for in vitro and in vivo experiments.

**Methods:** The peptides were labeled with gallium-67 as reported in the main article. It was observed that the commercial gallium-67 was not of sufficiently high quality to allow labeling of the peptides at high molar activity. ICP-MS analysis of one representative batch of gallium-67 revealed copper, zinc and iron metal impurities. After a post-purification process, the gallium-67 was obtained at high quality to allow the radiolabeling of the DX600-based peptides at reproducible molar activities. Quality control of the radiolabeled DX600-based peptides was performed after dilution in Milli-Q water containing sodium diethylenetriamine pentaacetic acid (Na_5_-DTPA; 50 μM) using a Merck Hitachi LaChrom HPLC system equipped with a D-7000 interface, a L-7200 autosampler, a radioactivity detector (LB 506 B; Berthold) and a L-7100 pump connected with a C18 column (Xterra^TM^, 5 μm, 4.6×150 mm, Waters, Milford, MA, U.S.A.). The radiopeptides were eluted using a linear gradient of Milli-Q water containing 0.1% TFA (95–20%) and acetonitrile (5–80%) over 15 min at a flow rate of 1.0 mL/min.

**Results:** The HPLC retention times were similar for all radiopeptides. Radiochemical purities of >98% were obtained, which allowed using the radiopeptides for subsequent in vitro and in vivo experimentations without further purification steps (Fig. S3).


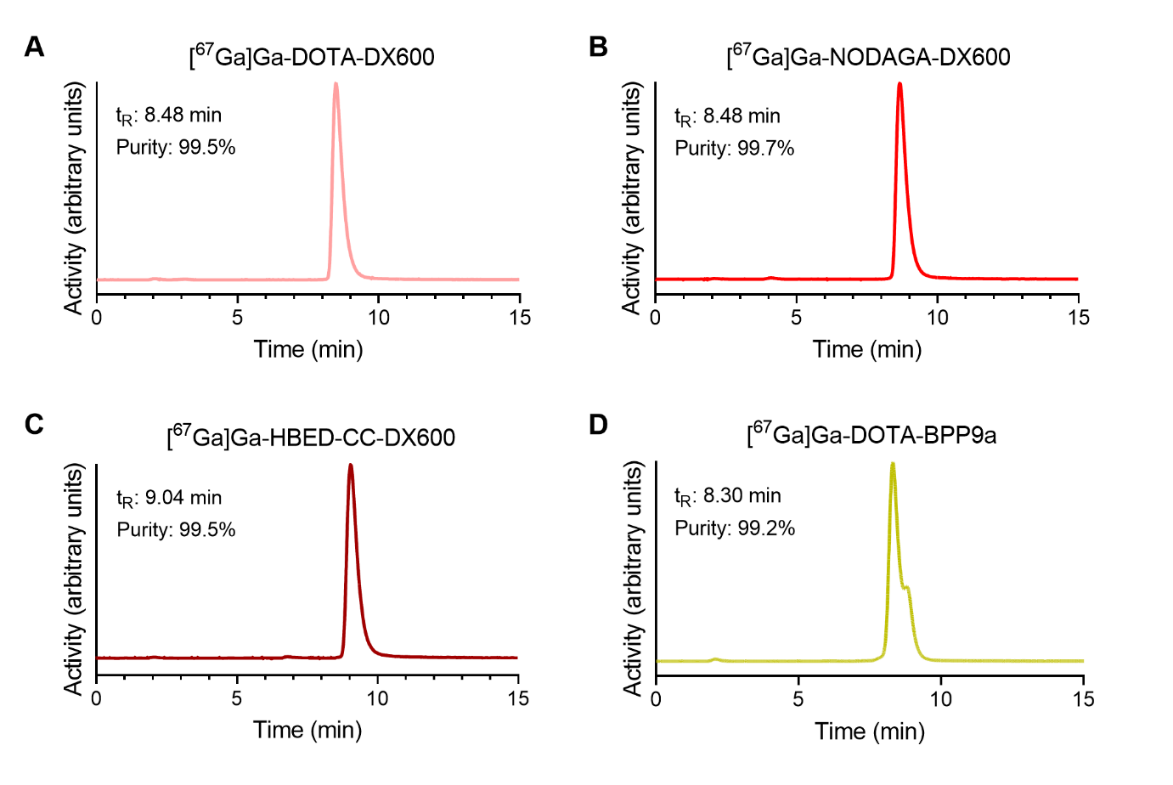


**Fig. S3** Representative chromatograms of the ^67^Ga-labeled peptides **A** [^67^Ga]Ga-DOTA-DX600, **B** [^67^Ga]Ga-NODAGA-DX600, **C** [^67^Ga]Ga-HBED-CC-DX600 and **D** [^67^Ga]Ga-DOTA-BPP9a. The retention times (t_R_) and radiochemical purities are indicated in the figures.

**4. Stability of the radiopeptide formulated in saline**

**Purpose:** The stability of the radiopeptides was determined for the highest activity concentration used for the herein described studies, which were the in vivo experiments.

**Methods:** The integrity of the radiopeptides (20 MBq/nmol), formulated in 0.9% NaCl (10 MBq/100 µL) and incubated at room temperature, was determined by HPLC using the system described above. The HPLC chromatograms were analyzed by determination of the peak area of the radiolabeled peptide, the released gallium-67, as well as degradation products of unknown structure. The quantity of the intact product was expressed as percentage of the sum of integrated peak areas of the entire chromatogram and set in relation to the original value determined at t = 0, which was set as 100%.

**Results:** [^67^Ga]Ga-DOTA-DX600, [^67^Ga]Ga-NODAGA-DX600 and [^67^Ga]Ga-HBED-CC-DX600 were stable over a 24 h period in saline. [^67^Ga]Ga-DOTA-BPP9a showed partial radiolysis at the 24 h-timepoint, however, it was found to be sufficiently stable over the time required for in vitro and in vivo experiments (Table S3).

**Table S3** Stability of radiopeptides determined after variable incubation periods, expressed as a percentage of the sum of integrated peak areas of the entire chromatogram, which was set as 100%.

| **Radiopeptide** | **Intact radiopeptide [%]** | | |
| --- | --- | --- | --- |
|  | 1 h | 3 h | 24 h |
| [^67^Ga]Ga-DOTA-DX600 | ≥99 | ≥99 | ≥99 |
| [^67^Ga]Ga-NODAGA-DX600 | ≥99 | ≥99 | ≥99 |
| [^67^Ga]Ga-HBED-CC-DX600 | ≥99 | ≥99 | ≥99 |
| [^67^Ga]Ga-DOTA-BPP9a | ≥99 | ≥99 | 89±1% |

**5. ^67^Ga-labeling efficiency of DX600-based peptides**

**Purpose:** The aim was to investigate at which molar activity ^67^Ga-labeling of the DX600-based peptides was still feasible in order to identify the best-suited chelator.

**Methods:** DOTA-DX600, NODAGA-DX600 and HBED-CC-DX600 were labeled with gallium-67 at a molar activity of 80 MBq/nmol as described above, followed by quality control using HPLC. Afterwards, more unlabeled peptide was added and the reaction mixture was heated again for 10 min to obtain a molar activity of 60 MBq/nmol followed by another quality control by HPLC. This process was repeated to obtain molar activities of 40 MBq/nmol and 20 MBq/nmol. The HPLC chromatograms were analyzed by determination of the peak area of the radiolabeled peptide, corresponding to the radiochemical purity.

**Results:** While the [^67^Ga]Ga-DOTA-DX600, labeled at a low molar activity of 20 MBq/nmol, was obtained with a radiochemical purity of >99%, a dramatic drop to <3% radiochemical purity was observed when using a molar activity of 40 MBq/nmol. NODAGA-DX600 performed slightly better than DOTA-DX600. At a molar activity of 20 MBq/nmol, [^67^Ga]Ga-NODAGA-DX600 was obtained with a radiochemical purity of >95%. When using HBED-CC-DX600, an almost 4-fold higher labeling efficiency was reached, resulting in >95% radiochemical purity at molar activities up to 60 MBq/nmol. Increasing the molar activity to 80 MBq/nmol resulted in a radiochemical purity of >81%. (Table S4).

**Table S4** Radiochemical purities obtained from the indicated [^67^Ga]Ga-radiopeptides with their respective molar activity.

|  | Radiochemical purity at variable molar activities | | | |
| --- | --- | --- | --- | --- |
| **Molar activity** | **20 MBq/nmol** | **40 MBq/nmol** | **60 MBq/nmol** | **80 MBq/nmol** |
| [^67^Ga]Ga-DOTA-DX600 | 99% | 3.0% | 1.3% | 0.3% |
| [^67^Ga]Ga-NODAGA-DX600 | 99% | 16% | 9.2% | 5.8% |
| [^67^Ga]Ga-HBED-CC-DX600 | 100% | 99% | 96% | 82% |

**6. Determination of the binding properties to mouse and human plasma proteins**

**Purpose**: The plasma protein-binding properties of the radiopeptides were investigated.

**Methods:** The detailed methods are reported in the main article. Control experiments were performed by incubation of the respective radiopeptides in phosphate-buffered saline (PBS) instead of blood plasma, followed by ultracentrifugation using Amicon filters.

**Results:** The DX600-based radiopeptides showed a comparable binding to both human and mouse blood plasma proteins (78–83% and 76–82%, respectively). [^67^Ga]Ga-DOTA-BPP9a showed lower plasma protein-binding properties (50% and 53%, in human and mouse plasma, respectively; Fig. S4). The unspecific binding determined in control experiments was low for all radiopeptides (<15%), indicating moderate adherence to the filter surface.

**
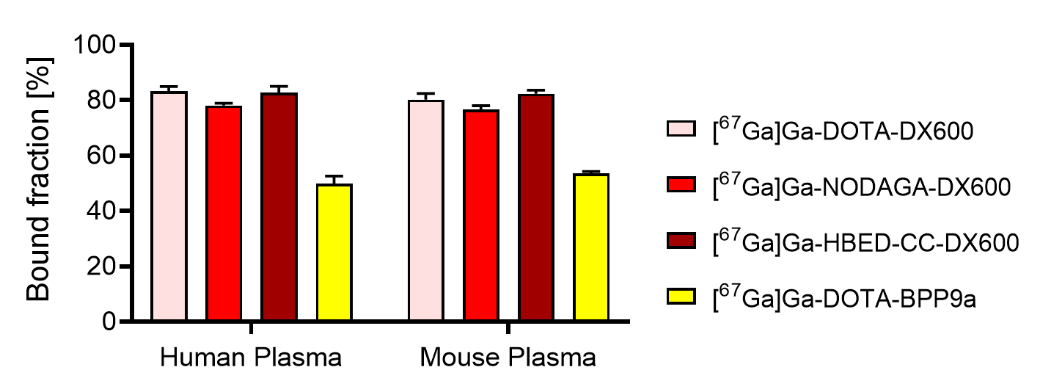
**

**Fig. S4** Binding of the radiopeptides to blood plasma proteins using mouse and human blood plasma.

**7. Western blot analysis of HEK-ACE2 and HEK-ACE cells**

**Purpose:** Western blot analysis was performed with HEK-ACE2 and HEK-ACE cells to verify the selective expression of each enzyme on these cell lines.

**Methods:** The cells (3–4 x 10^6^ cells) were seeded into 6 cm-Petri dishes using complete culture medium and let to grow overnight. Cell lysates were prepared using 150 µL cell lysis buffer (Cell Signaling Technology), with a protease inhibitor cocktail (cOmplete^TM^, Roche) and phosphatase inhibitor cocktail 3 (Merck). The lysates were centrifuged (10 min, 16100 rcf, 4 °C) to get rid of cell debris. The protein concentration of the cell lysates was determined using Coomassie Plus Protein Reagent. The samples were mixed with loading buffer containing dithiothreitol and mercaptoethanol before separation by sodium dodecyl sulfate polyacrylamide gel electrophoresis (SDS-PAGE). Proteins were transferred to a polyvinylidene fluoride membrane with a semi-dry blotting device (BioRad Laboratories, Reinach Switzerland). Skim milk (5%) in Tris-buffered saline containing 0.05% Tween 20 (TBST, pH 7.5) was used to prevent unspecific binding of the antibody. Incubation with monoclonal IgG anti-ACE2 antibody (Santa Cruz Biotechnology, E11, sc-390851; diluted 1:1500) or monoclonal IgG anti-ACE antibody (Santa Cruz Biotechnology, 2E2, sc-23908; diluted 1:1000) was performed overnight at 4 °C in TBST containing 2% BSA. A secondary anti-mouse IgG antibody (Cell Signaling Technology, #7076; diluted 1:3000) functionalized with horseradish peroxidase (HRP) was used together with Amersham Prime, ECL Western Blotting Detection Reagent for signal detection. Detection of glyceraldehyde-3-phosphate-dehydrogenase (GAPDH) using rabbit monoclonal anti-GAPDH antibody (Cell Signaling Technology, #5174, diluted 1:2000) and HRP-linked, anti-rabbit IgG (Cell Signaling Technology, #7074; diluted 1:5000), served as a loading control.

**Results:** The resulting Western blots demonstrated a positive signal for HEK-ACE2 cells when using the anti-ACE2 antibody but no signal for the HEK-ACE cells (Fig. S5A). In contrast, no signal was observed on HEK-ACE2 cells when using an anti-ACE antibody, however, a clear signal was obtained for HEK-ACE cells (Fig. S5B).


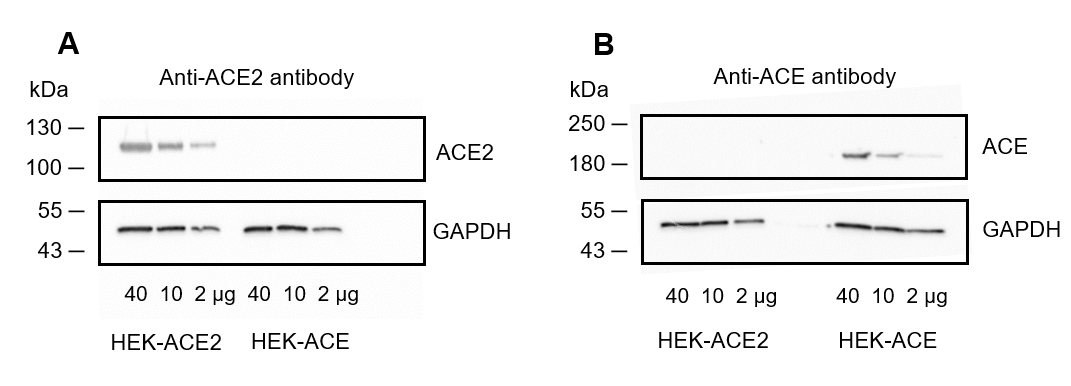


**Fig. S5** Western blot obtained using lysates (40 μg, 10 μg and 2 μg) of HEK-ACE2 and HEK-ACE cells. **A** Protein staining with anti-ACE2. **B** Protein staining with anti-ACE.

**8. Receptor-specific binding of the radiopeptides in vitro**

**Purpose:** The question about receptor-specific binding of the DX600-based radiopeptides and [^67^Ga]Ga-DOTA-BPP9a was assessed using HEK-ACE2 and HEK-ACE cells, respectively.

**Methods:** The methods for cell uptake studies are reported in the main article. The cyclo-DX600 (ACE2 inhibitor) and lisinopril (ACE inhibitor) were applied at a final concentration of 2 µM and 4 µM, respectively.

**Results:** The ACE2-specific uptake of the DX600-based radiopeptides was confirmed by the fact that their uptake of up to 43% in HEK-ACE2 cells was almost entirely blocked (<3% uptake) if the cells were co-incubated with excess cyclo-DX600 (Fig. S6A). The uptake of [^67^Ga]Ga-DOTA-BPP9a (16±2%) in HEK-ACE cells was entirely blocked (<1% uptake) when using an excess of lisinopril which confirmed the ACE-specific uptake of this radiopeptide (Fig. S6B).

**
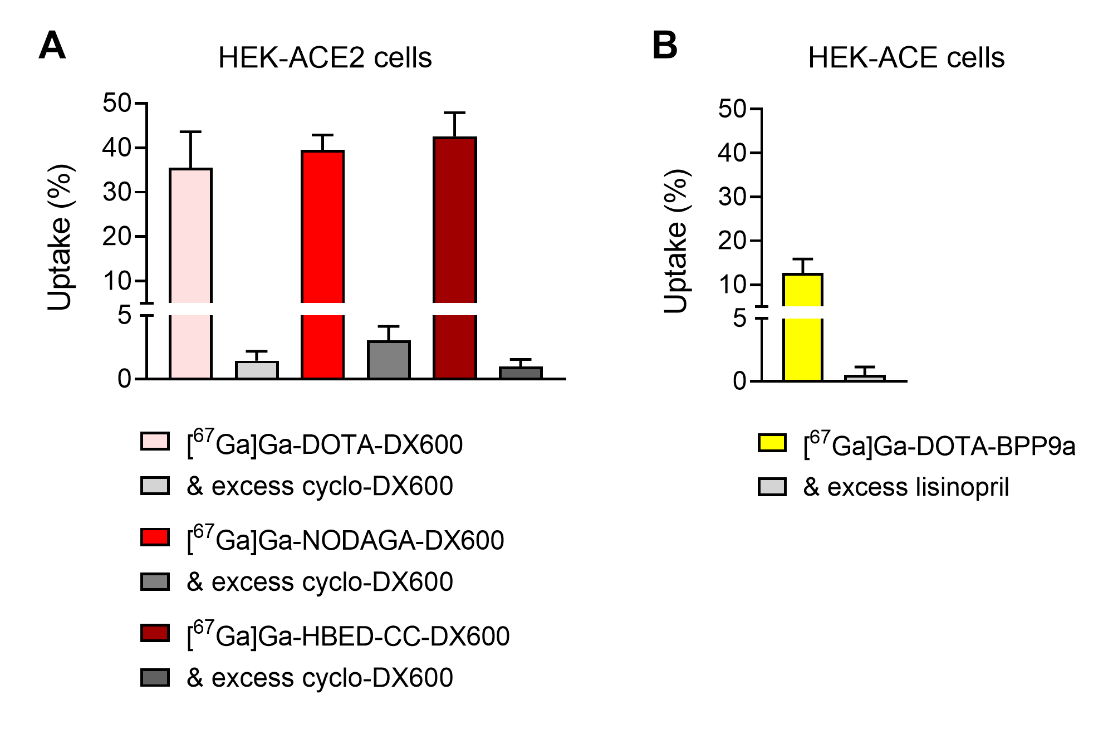
**

**Fig. S6** Cell uptake of the radiopeptides in the absence and presence of specific inhibitors of the respective receptor. **A** HEK-ACE2 incubated with the DX600-based radiopeptides in the absence and presence of an excess of the ACE-2-specific inhibitor cyclo-DX600. **B** HEK-ACE cells incubated with [^67^Ga]Ga-DOTA-BPP9a in the absence and presence of the ACE-specific inhibitor lisinopril.

**9. Blood plasma stability of DX600-based radiopeptides**

**Purpose:** The DX600-based radiopeptides were investigated after in vitro incubation in mouse and human blood plasma to investigate potential enzymatic degradation.

**Methods:** The integrity of the radiopeptides (20 MBq/nmol) in mouse and human blood plasma (Rockland and Blood donation SRK Aargau-Solothurn, respectively) was determined using thin layer chromatography (TLC). Reversed phase C-18 plates (TLC Silica gel 60 RP-18; Merck) were used as the stationary phase and citrate buffer (pH 5.5; 0.1 M) as the mobile phase. Under these conditions, uncoordinated gallium-67 as well as chelated gallium-67 migrated, while radiolabeled DX600-peptides stayed at the baseline. The data obtained using this method were confirmed with normal phase silica gel plates (TLC Silica gel 60; Merck) as the stationary phase and a mixture of ammonium acetate (10%) and methanol (1:1, v/v) as the mobile phase. Under these conditions, uncoordinated gallium-67 stayed at the baseline while the radiopeptides and potential larger fragments migrated.

The radiopeptides were prepared and TLC experiments were performed immediately after radiolabeling by applying the diluted radiopeptides in saline (1 µL; ~0.05 MBq) and [^67^Ga]GaCl_3_ solution (1 µL; ~0.05 MBq) on the TLC plates. The radiopeptides were incubated in mouse or human plasma (10 MBq/200 µL) at 37° C. TLC was performed with an aliquot of the plasma samples at the given timepoints (30 min, 1 h, 3 h and 24 h). The TLC plates were exposed to a phosphor screen followed by development using a storage phosphor system (Cyclone Plus, PerkinElmer). The quantification of the signals was carried out using the OptiQuant software (version 5.0, Bright Instrument Co Ltd., PerkinElmer^TM^). The obtained chromatograms were analyzed by determination of the peak area of the radiolabeled peptide, the released gallium-67 as well as degradation products of unknown structure. The quantity of the intact product was expressed as percentage of the sum of integrated peak areas of the entire chromatogram.

**Results:** The results are described in the main article and shown below (Fig. S7).


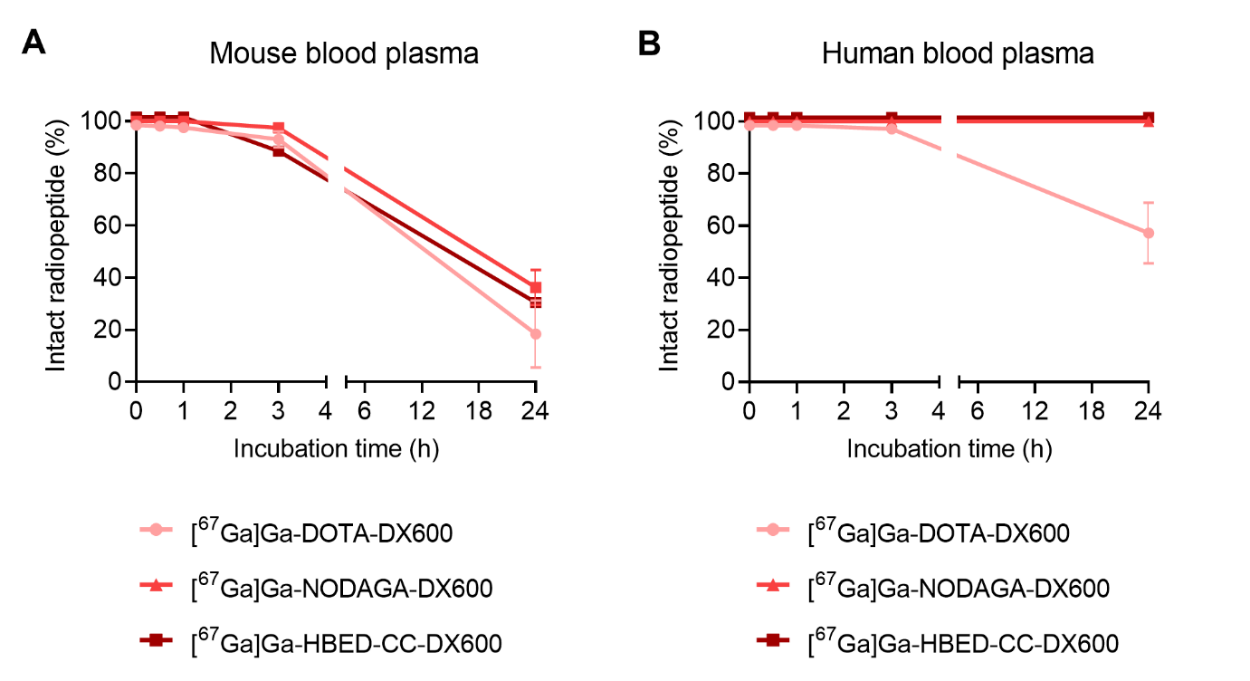


**Fig. S7** Graphs indicating the intact fraction of radiopeptides incubated in mouse or human blood plasma. **A** Intact fraction of radiopeptides incubated in mouse plasma. **B** Intact fraction of radiopeptides incubated in human plasma.

**10. Biodistribution studies in HEK-ACE2/HEK-ACE xenografted mice**

**Purpose:** Biodistribution studies were performed in CD1/nude mice bearing HEK-ACE2 and HEK-ACE xenografts on the right and left shoulder, respectively. The aim was to investigate whether the radiopeptides showed specific accumulation in ACE2-expressing xenografts and not in ACE-expressing xenografts.

**Methods:** The methods are reported in the main article.

**Results:** The biodistribution data are reported in the main article and listed in Table S5.

**Table S5** Decay-corrected biodistribution data obtained 3 h after injection of the radiopeptides in HEK-ACE2/HEK-ACE-xenografted mice. The values are indicated as the average ± SD obtained from each group of mice (n = 3) and listed as the percentage of injected activity per gram of tissue [% IA/g].

|  | **[^67^Ga]Ga-DOTA-**  **DX600** | **[^67^Ga]Ga-NODAGA-**  **DX600** | **[^67^Ga]Ga-HBED-CC-**  **DX600** | **[^67^Ga]Ga-DOTA-**  **BPP9a** |
| --- | --- | --- | --- | --- |
| blood | 0.06 ± 0.01 | 0.02 ± 0.01 | 0.09 ± 0.04 | 0.05 ± 0.01 |
| heart | 0.04 ± 0.01 | 0.04 ± 0.01 | 0.07 ± 0.03 | 0.03 ± 0.00 |
| lung | 0.14 ± 0.06 | 0.08 ± 0.01 | 0.13 ± 0.05 | 0.13 ± 0.03 |
| spleen | 0.09 ± 0.01 | 0.08 ± 0.01 | 0.06 ± 0.03 | 0.08 ± 0.04 |
| kidneys | 24.0 ± 0.8 | 23.9 ± 4.5 | 7.2 ± 2.2 | 3.22 ± 0.80 |
| stomach | 0.04 ± 0.01 | 0.06 ± 0.03 | 0.09 ± 0.03 | 0.06 ± 0.05 |
| intestines | 0.09 ± 0.04 | 0.13 ± 0.07 | 0.43 ± 0.31 | 0.06 ± 0.02 |
| liver | 0.22 ± 0.01 | 0.25 ± 0.04 | 0.23 ± 0.16 | 0.09 ± 0.01 |
| muscle | 0.02 ± 0.00 | 0.02 ± 0.01 | 0.03 ± 0.02 | 0.02 ± 0.01 |
| bone | 0.06 ± 0.01 | 0.04 ± 0.01 | 0.05 ± 0.02 | 0.04 ± 0.01 |
| salivary glands | 0.05 ± 0.01 | 0.05 ± 0.01 | 0.05 ± 0.02 | 0.04 ± 0.01 |
| ACE2 xenograft | 10.7 ± 0.4 | 15.5 ± 2.2 | 12.3 ± 1.3 | 0.19 ± 0.19 |
| ACE xenograft | 0.32 ± 0.18 | 0.22 ± 0.17 | 0.27 ± 0.33 | 3.17 ± 0.29 |

**11. SPECT/CT imaging studies**

**Purpose:** SPECT/CT imaging studies in CD1/nude mice bearing a HEK-ACE2 and a HEK-ACE xenograft on the right and left shoulder, respectively, were performed in order to visualize the distribution of the DX600-based radiopeptides. SPECT imaging was performed after injection of [^67^Ga]Ga-DOTA-BPP9a in order to validate the mouse model in terms of ACE-expressing xenograft.

**Methods:** The detailed methods of the SPECT/CT acquisitions are reported in the main article.

**Results:** The SPECT/CT images showed no uptake of [^67^Ga]Ga-DOTA-BPP9a in the HEK-ACE2 xenografts but accumulation in the HEK-ACE xenografts. Significant retention of this radiopeptide was also found in the kidneys (Fig. S8).


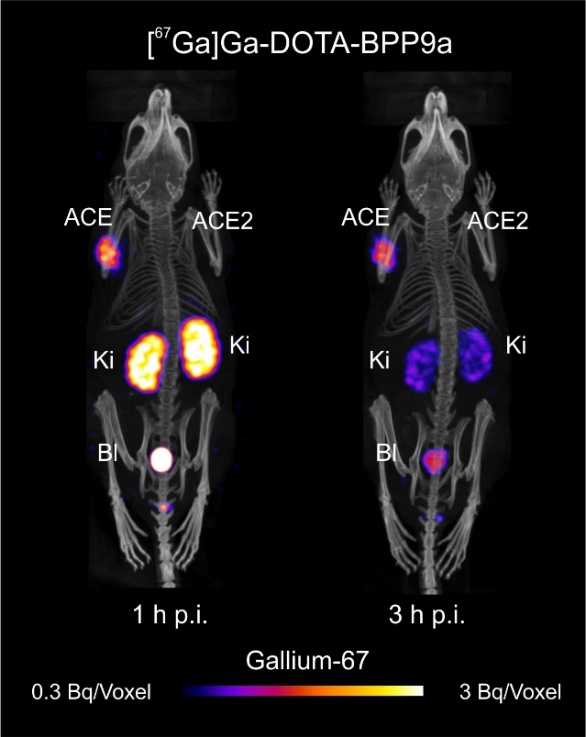


**Fig. S8** SPECT/CT images of a HEK-ACE2/HEK-ACE xenografted mouse acquired 1 h and 3 h after injection of [^67^Ga]Ga-DOTA-BPP9a.

**12. In vivo stability of DX600-based radiopeptides**

**Purpose:** One hour after injection of the DX600-based radiopeptides into immunocompetent mice, blood plasma, urine and kidneys were investigated for the presence of the intact radiopeptide and potential radiometabolite formation.

**Methods:** Immunocompetent FVB mice were injected with the DX600-based radiopeptides (n=2). One hour later, urine was collected and blood was sampled from the retrobulbar vein followed by centrifugation (10 min, 200 rcf). A drop of urine and blood plasma was analyzed using the TLC method described for in vitro stability experiments above (reversed phase as stationary phase and citrate buffer pH 5.5 as mobile phase). The data were also confirmed using normal phase TLC. Kidneys of mice were collected, cut into small pieces, homogenized in 200 μL buffer solution (ice-cold methanol containing 0.025% NH_4_OH) and centrifuged (5 min, 2300 rcf, 4° C) as described by Boss et al. [3]. An aliquot of the supernatant of each sample was developed using the same TLC method as for urine and blood plasma samples. The TLC plates were exposed to a phosphor screen which was afterwards read using a storage phosphor system (Cyclone Plus, PerkinElmer). The quantification of the signals was carried out using the OptiQuant software (version 5.0, Bright Instrument Co Ltd., PerkinElmer^TM^). The obtained chromatograms were analyzed by determination of the peak area of the radiolabeled peptide, the released gallium-67 as well as degradation products of unknown structure. The quantity of the intact product was expressed as percentage of the sum of integrated peak areas of the entire chromatogram.

**Results:** The results are described in the main article and shown below (Fig. S9).

**
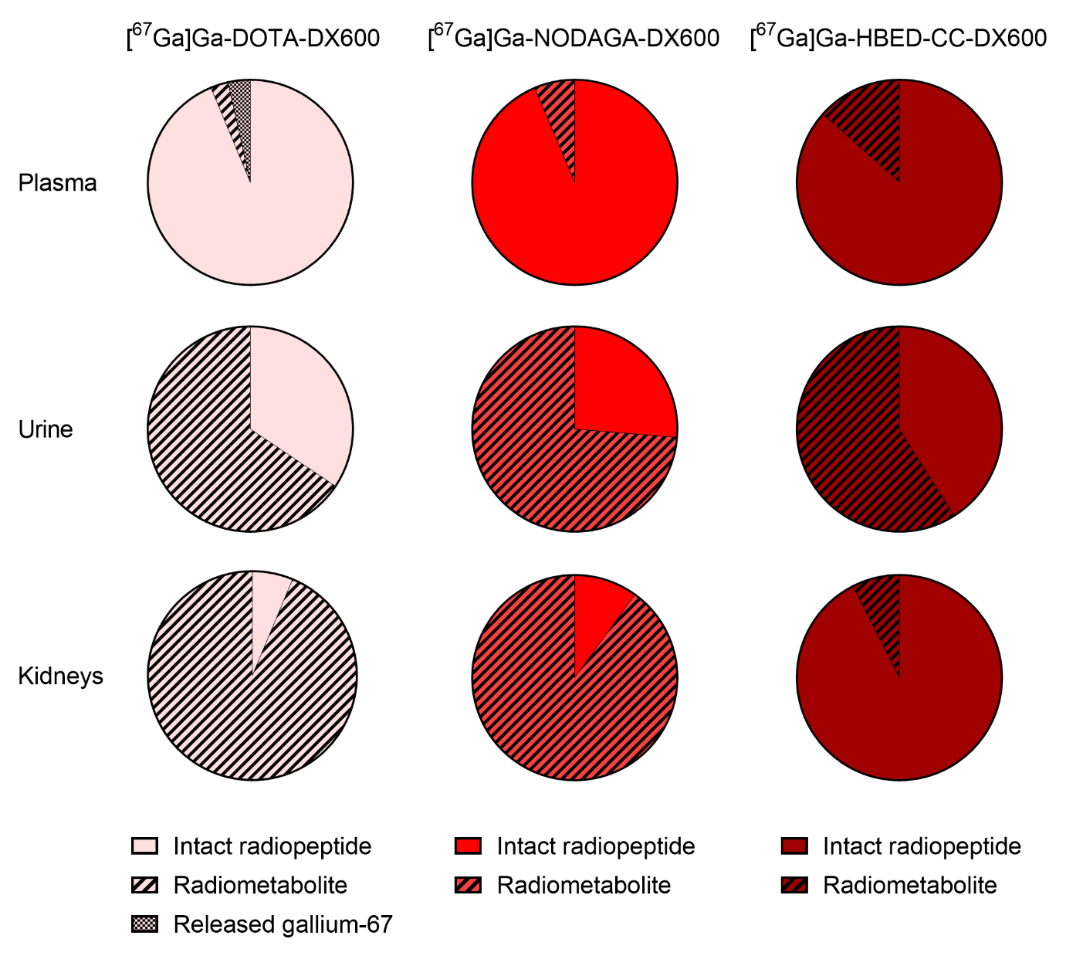
**

**Fig. S9** Diagrams showing the fraction of intact DX600-based radiopeptides (plain color), radiometabolites (dashed) in samples of blood plasma, urine and the kidneys at 1 h after injection. In the case of [^67^Ga]Ga-DOTA-DX600 in blood plasma, a small fraction was identified as released gallium-67 (dotted).

**13. In vitro autoradiography**

**Purpose:** In vitro autoradiography was performed on frozen tissue sections of HEK-ACE2 and HEK-ACE xenografts to demonstrate the effect of variable molar activities of [^67^Ga]Ga-HBED-CC-DX600 on the receptor binding and saturation, respectively.

**Methods:** The methods are reported in the main article.

**Results:** No visible differences in signal intensity were observed for HEK-ACE2 xenograft tissue sections exposed to the radiopeptide at molar activities higher than 5 MBq/nmol. Application of the radiopeptide prepared at lower molar activities (0.1–1 MBq/nmol) affected the radiopeptide's tissue binding due to saturation effects caused by the unlabeled fraction of the peptide. Moreover, the addition of an excess of cyclo-DX600 to the incubation solution almost completely prevented the binding of [^67^Ga]Ga-HBED-CC-DX600. The signal intensity of HEK-ACE xenograft sections was only in the range of background signal (Fig. S10). The signal intensity of each setting was expressed in comparison to the signal intensity of the highest signal (set as 100%) obtained for the sections exposed to the radiopeptide prepared at 20 MBq/nmol. The quantification data represent the average of three independent experiments.

**
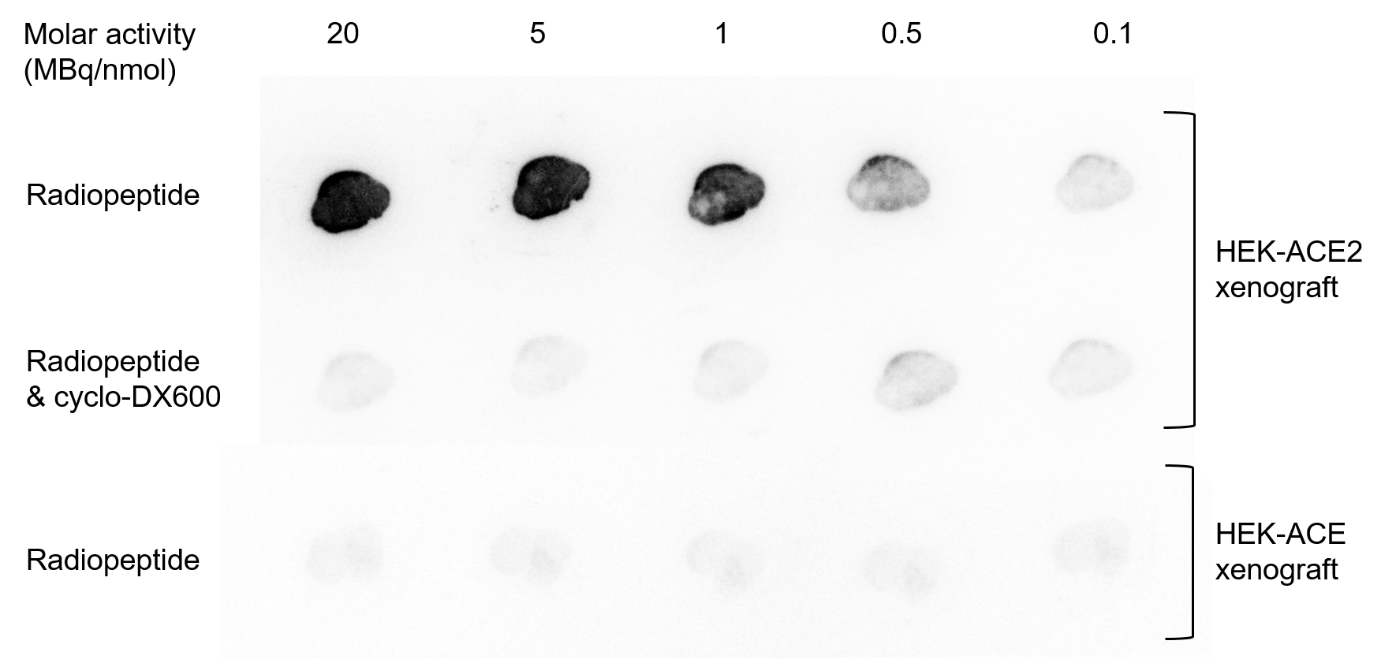
**

**Fig. S10** Representative autoradiograms obtained upon exposure of HEK-ACE2 and HEK-ACE xenograft sections to [^67^Ga]Ga-HBED-CC-DX600 prepared at variable molar activities. Tissue sections of HEK-ACE2 xenografts were incubated with [^67^Ga]Ga-HBED-CC-DX600 at different molar activities with and without an excess of cyclo-DX600 (10 µM). Tissue sections of HEK-ACE xenografts were incubated with the same radiopeptide solutions but no blocking experiments were performed with an excess of cyclo-DX600.

**References**

1. Ondetti MA, Williams NJ, Sabo EF, Pluscec J, Weaver ER, Kocy O. Angiotensin-converting enzyme inhibitors from the venom of Bothrops jararaca. Isolation, elucidation of structure, and synthesis. Biochemistry. 1971;10:4033-9. doi:10.1021/bi00798a004.

2. Gesquiere JC, Diesis E, Cung MT, Tartar A. Slow isomerization of some proline-containing peptides inducing peak splitting during reversed-phase high-performance liquid-chromatography. J Chromatogr. 1989;478:121-9. doi:Doi 10.1016/0021-9673(89)90010-1.

3. Boss SD, Müller C, Siwowska K, Schmid RM, Groehn V, Schibli R, et al. Diastereomerically pure 6R- and 6S-3'-aza-2'-^18^F-fluoro-5-methyltetrahydrofolates show unprecedentedly high uptake in folate receptor-positive KB tumors. J Nucl Med. 2019;60:135-41. doi:10.2967/jnumed.118.213314.
